# IMI2-PainCare-BioPain-RCT3: a randomized, double-blind, placebo-controlled, crossover, multi-center trial in healthy subjects to investigate the effects of lacosamide, pregabalin, and tapentadol on biomarkers of pain processing observed by electroencephalography (EEG)

**DOI:** 10.1186/s13063-021-05272-y

**Published:** 2021-06-17

**Authors:** André Mouraux, Petra Bloms-Funke, Irmgard Boesl, Ombretta Caspani, Sonya C. Chapman, Giulia Di Stefano, Nanna Brix Finnerup, Luis Garcia-Larrea, Marcus Goetz, Anna Kostenko, Bernhard Pelz, Esther Pogatzki-Zahn, Karin Schubart, Alexandre Stouffs, Andrea Truini, Irene Tracey, Iñaki F. Troconiz, Johannes Van Niel, Jose Miguel Vela, Katy Vincent, Jan Vollert, Vishvarani Wanigasekera, Matthias Wittayer, Keith G. Phillips, Rolf-Detlef Treede

**Affiliations:** 1 Institute of Neuroscience (IoNS), UCLouvain, Brussels, Belgium; 2 Translational Science & Intelligence, Grünenthal GmbH, Aachen, Germany; 3 Clinical Science Development, Grünenthal GmbH, Aachen, Germany; 4 Department of Neurophysiology, Medical Faculty Mannheim, University of Heidelberg, Mannheim, Germany; 5 Eli Lilly and Company, Erl Wood, UK; 6 Department of Human Neuroscience, Sapienza University, Rome, Italy; 7 Danish Pain Research Center, Department of Clinical Medicine, Aarhus University, Aarhus, Denmark; 8 Lyon Neurosciences Center Research Unit Inserm U 1028, Pierre Wertheimer Hospital, Hospices Civils de Lyon, Lyon 1 University, Lyon, France; 9 MRC Systems GmbH, Heidelberg, Germany; 10 Department of Anaesthesiology, Intensive Care and Pain Medicine, University Hospital Münster, Münster, Germany; 11 Consultech GmBH, Berlin, Germany; 12 Wellcome Centre for Integrative Neuroimaging, Nuffield Department of Clinical Neurosciences, University of Oxford, Oxford, UK; 13 Department of Pharmaceutical Technology and Chemistry, School of Pharmacy and Nutrition, University of Navarra, Pamplona, Spain; 14 Mature Products Development, Grünenthal GmbH, Aachen, Germany; 15 Drug Discovery & Preclinical Development, ESTEVE Pharmaceuticals, Barcelona, Spain; 16 Nuffield Department of Women’s and Reproductive Health (NDWRH), University of Oxford, Oxford, UK; 17 Pain Research, Department of Surgery and Cancer, Imperial College London, London, UK

**Keywords:** Pain, Analgesics, PK/PD, EEG, Biomarkers, Laser-evoked potentials, Pinprick-evoked potentials, Hyperalgesia, Randomized controlled trial, Healthy subjects

## Abstract

**Background:**

IMI2-PainCare-BioPain-RCT3 is one of four similarly designed clinical studies aiming at profiling a set of functional biomarkers of drug effects on the nociceptive system that could serve to accelerate the future development of analgesics, by providing a quantitative understanding between drug exposure and effects of the drug on nociceptive signal processing in human volunteers. IMI2-PainCare-BioPain-RCT3 will focus on biomarkers derived from non-invasive electroencephalographic (EEG) measures of brain activity.

**Methods:**

This is a multisite single-dose, double-blind, randomized, placebo-controlled, 4-period, 4-way crossover, pharmacodynamic (PD) and pharmacokinetic (PK) study in healthy subjects. Biomarkers derived from scalp EEG measurements (laser-evoked brain potentials [LEPs], pinprick-evoked brain potentials [PEPs], resting EEG) will be obtained before and three times after administration of three medications known to act on the nociceptive system (lacosamide, pregabalin, tapentadol) and placebo, given as a single oral dose in separate study periods. Medication effects will be assessed concurrently in a non-sensitized normal condition and a clinically relevant hyperalgesic condition (high-frequency electrical stimulation of the skin). Patient-reported outcomes will also be collected. A sequentially rejective multiple testing approach will be used with overall alpha error of the primary analysis split between LEP and PEP under tapentadol. Remaining treatment arm effects on LEP or PEP or effects on EEG are key secondary confirmatory analyses. Complex statistical analyses and PK-PD modeling are exploratory.

**Discussion:**

LEPs and PEPs are brain responses related to the selective activation of thermonociceptors and mechanonociceptors. Their amplitudes are dependent on the responsiveness of these nociceptors and the state of the pathways relaying nociceptive input at the level of the spinal cord and brain. The magnitude of resting EEG oscillations is sensitive to changes in brain network function, and some modulations of oscillation magnitude can relate to perceived pain intensity, variations in vigilance, and attentional states. These oscillations can also be affected by analgesic drugs acting on the central nervous system. For these reasons, IMI2-PainCare-BioPain-RCT3 hypothesizes that EEG-derived measures can serve as biomarkers of target engagement of analgesic drugs for future Phase 1 clinical trials. Phase 2 and 3 clinical trials could also benefit from these tools for patient stratification.

**Trial registration:**

This trial was registered 25/06/2019 in EudraCT (2019%2D%2D001204-37).

## Administrative information

The order of the items has been modified to group similar items (see http://www.equator-network.org/reporting-guidelines/spirit-2013-statement-defining-standard-protocol-items-for-clinical-trials/).

**Table:** 

Title {1}	IMI2-PainCare-BioPain-RCT3: A randomized, double-blind, placebo-controlled, cross-over, multi-center trial in healthy subjects to investigate the effects of lacosamide, pregabalin and tapentadol on biomarkers of pain processing observed by electro-encephalography (EEG)
Trial registration {2a and 2b}.	EudraCT registration: 2019–001204-37
Protocol version {3}	3.0 (15/05/2019)
Funding {4}	This project has received funding from the Innovative Medicines Initiative 2 Joint undertaking under grant agreement No 777500. This Joint Undertaking receives support from the European Union’s Horizon 2020 research and innovation program and EFPIA.
Author details {5a}	André Mouraux^1^, Petra Bloms-Funke^2^, Irmgard Boesl^3^, Ombretta Caspani^4^, Sonya C Chapman^5^, Giulia Di Stefano^6^, Nanna Brix Finnerup^7^, Luis Garcia-Larrea^8^, Marcus Goetz^9^, Anna Kostenko^4^, Bernhard Pelz^9^, Esther Pogatzki-Zahn^10^, Karin Schubart^11^, Alexandre Stouffs^1^, Andrea Truini^6^, Irene Tracey^12^, Iñaki F. Troconiz^13^, Hans Van Niel^14^, Jose Miguel Vela^15^, Katy Vincent^16^, Jan Vollert^17^, Vishvarani Wanigasekera^12^, Matthias Wittayer^4^, Keith G Phillips^5^*, Rolf-Detlef Treede^4^* 1. Institute of Neuroscience (IoNS), UCLouvain, Brussels, Belgium. 2. Translational Science & Intelligence, Grünenthal GmbH, Aachen, Germany. 3. Clinical Science Development, Grünenthal GmbH, Aachen, Germany. 4. Department of Neurophysiology, Medical Faculty Mannheim, University of Heidelberg, Mannheim, Germany. 5. Eli Lilly and Company, Erl Wood, UK 6. Department of Human Neuroscience, Sapienza University, Rome, Italy. 7. Danish Pain Research Center, Department of Clinical Medicine, Aarhus University, Aarhus, Denmark. 8. Lyon Neurosciences Center Research Unit Inserm U 1028, Pierre Wertheimer Hospital, Hospices Civils de Lyon, Lyon 1 University, Lyon, France. 9. MRC Systems GmbH, Heidelberg, Germany. 10. Department of Anaesthesiology, Intensive Care and Pain Medicine, University Hospital Münster, Münster, Germany. 11. Consultech GmbH, Berlin, Germany 12. Wellcome Centre for Integrative Neuroimaging, Nuffield Department of Clinical Neurosciences, University of Oxford, Oxford, United Kingdom 13. Department of Pharmaceutical Technology and Chemistry, School of Pharmacy and Nutrition, University of Navarra, Pamplona, Spain. 14. Mature Products Development, Grünenthal GmbH, Aachen, Germany. 15. Drug Discovery & Preclinical Development, ESTEVE Pharmaceuticals, Barcelona, Spain. 16. Nuffield Department of Women’s and Reproductive Health (NDWRH), University of Oxford, Oxford, UK. 17. Pain Research, Department of Surgery and Cancer, Imperial College London, London, UK.
Name and contact information for the trial sponsor {5b}	André Mouraux, Institute of Neuroscience (IoNS), UCLouvain, 53 Avenue Mounier, B1200, Brussels, Belgium. Email: andre.mouraux@uclouvain.be. Tel: + 32(0)2764 54 47
Role of sponsor {5c}	This study is one of four studies conducted in subtopic BioPain of the IMI2-PainCare project, coordinated by Rolf-Detlef Treede (Heidelberg University). Design of the study was led by the sponsor and coordinator, and involved all partners of the BioPain subtopic of the IMI2-PainCare consortium (WP5, WP6, WP7). The sponsor will coordinate data collection at all sites and extract all biomarker parameters from the source data. Final analysis of study endpoints will be coordinated by Rolf-Detlef Treede and involve all partners of the BioPain subtopic of the IMI2-PainCare consortium.

## Introduction

### Background and rationale {6a}

Currently available pharmacological therapies provide inadequate relief for many patients with chronic pain. It is known that novel drugs which are efficacious analgesics in preclinical models often have little or no clinical efficacy, but it is often not known whether the drug engaged the human target sufficiently to have a meaningful pharmacodynamic effect. Hence, early deselection of unpromising candidates would greatly reduce attrition rates in clinical development. This was encouraged by the recently revised EMA/CHMP Guideline on the clinical development of medicinal products intended for the treatment of pain (EMA/CHMP/970057/2011). We postulate that this could be achieved by using a novel research paradigm taking advantage of improved objective measures of nociceptive signal processing, functional biomarkers of pain which translate between animals and humans, and pharmacokinetic-pharmacodynamic (PK-PD) modeling.

The overall concept of the BioPain subtopic of IMI-PainCare (http://imi-paincare.eu) is that psychophysical, electrophysiological, and neuroimaging biomarkers can be used to build a quantitative understanding (i) between drug exposure and target engagement (proof of mechanism) and (ii) between target engagement and effect on pathophysiology (proof of principle and/or concept) in both animals and its extrapolation to humans. By identifying specific mechanisms within pain pathways in healthy volunteers, these same quantitative neurophysiological biomarkers have the opportunity—if well validated in patients—to permit patient stratification and enrichment in later clinical trials as encouraged by the EMA/CHMP/970057/2011 Guideline. This could accelerate the development of novel analgesics in several ways: preclinical prediction could be improved by using translatable readouts across species; clinical Phase 1 trials could benefit from biomarkers of target engagement and from human surrogate models predictive of clinical efficacy; clinical Phase 2 and 3 studies could benefit from tools for patient stratification. BioPain will analyze these functional pain biomarkers in healthy subjects and in preclinical species, where they will also be compared with standard behavioral assessments.

### Objectives {7}

BioPain hypothesizes that a set of functional biomarkers of the effects of analgesic drugs on nociceptive processing at peripheral, spinal, and supraspinal level can be derived from non-invasive electrophysiological and psychophysical measures of peripheral nociceptor activity, electrophysiological measures of spinal and brainstem reflex activity, and electrophysiological/functional magnetic resonance imaging measures of brain activity.

For this purpose, BioPain has designed four placebo-controlled RCTs in healthy subjects with the objective of profiling four sets of pain biomarkers derived from non-invasive measures of peripheral nerve excitability (IMI2-PainCare-BioPain-RCT1),^1^, electrophysiological measures of spinal cord and brainstem reflex activity (IMI2-PainCare-BioPain-RCT2),^2^ electroencephalographic (EEG) measures of brain activity (IMI2-PainCare-BioPain-RCT3),^3^ and functional magnetic resonance imaging measures of brain activity (IMI2-PainCare-BioPain-RCT4),^4^ using three drugs registered as analgesics or known to act on the nociceptive system, given as a single dose in four separate study periods: lacosamide acting preferentially on nociception at peripheral level, pregabalin acting preferentially on nociception at the spinal level, and tapentadol acting preferentially on nociception at the supraspinal level.

IMI2-PainCare-BioPain-RCT3 will thus focus on biomarkers derived from non-invasive EEG measurements. Specifically, it will evaluate laser-evoked brain potentials (LEPs) which are brain responses related to the selective activation of heat-sensitive cutaneous nociceptors [1–3], pinprick-evoked brain potentials (PEPs) which are brain responses related to the preferential activation of mechano-sensitive nociceptors [4, 5], and ongoing EEG whose oscillations have been shown to be sensitive to changes in brain network function, to relate to perceived pain intensity, to correlate with variations in vigilance and attentional states, and to be sensitive to the effects of several analgesic drugs acting at the level of the central nervous system [6–8].

Crucially, all four studies of the BioPain project will assess the effects of the medications on the biomarkers concurrently in a non-sensitized normal condition and a clinically relevant hyperalgesic condition. For this purpose, the trials will use high-frequency electrical stimulation (HFS) of the skin, a validated and non-invasive experimental procedure to induce, in healthy volunteers, a reversible but nevertheless sustained state of hyperalgesia due to sensitization of the nociceptive system [9, 10].

### Trial design {8}

IMI2-PainCare-BioPain-RCT3 is a placebo-controlled randomized trial (RCT) conducted in healthy subjects and designed to profile a set of pain biomarkers derived from non-invasive electroencephalographic (EEG) measures of brain activity.

**Fig. 1:**
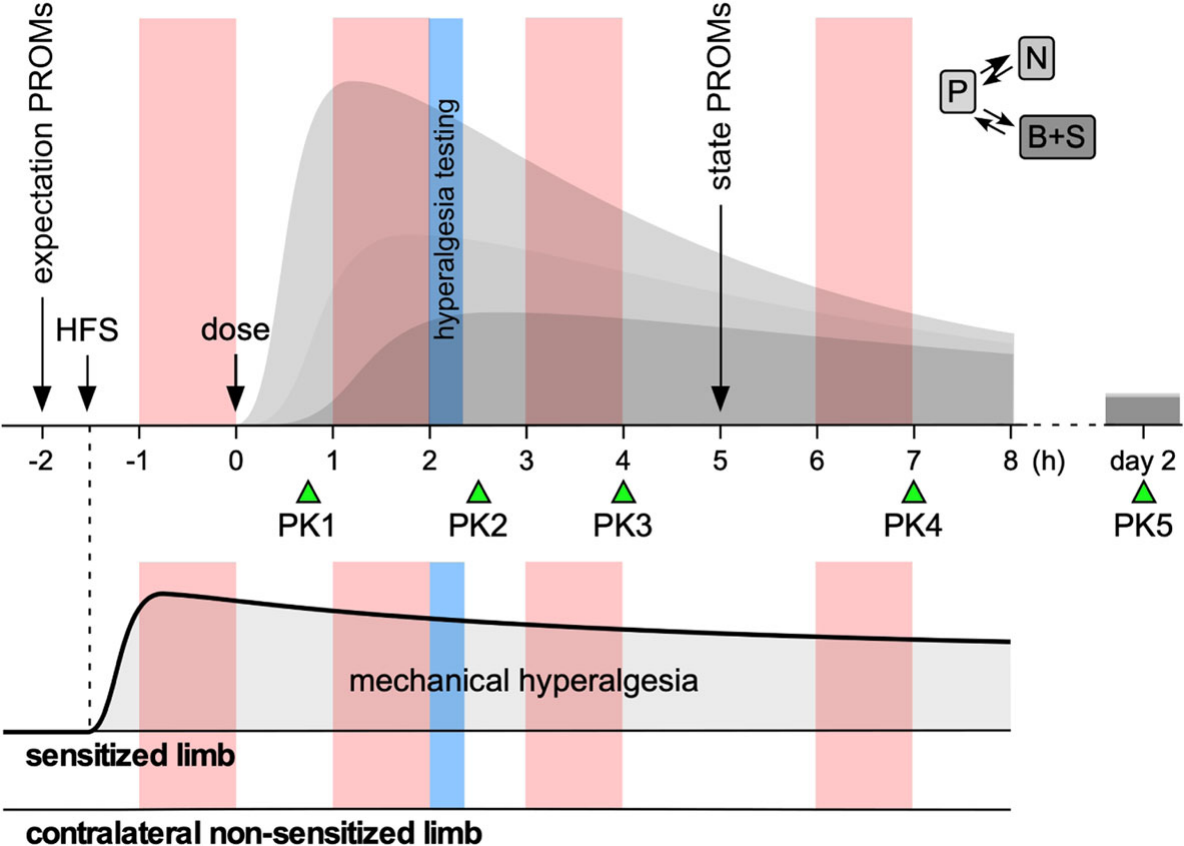
Trial design. The effects of a single oral dose of four different treatments (lacosamide, pregabalin, tapentadol, placebo) on EEG-derived biomarkers of nociceptive processing will be assessed in four separate study periods separated by at least 1 week. In each study period, five blood samples will be taken to model the pharmacokinetic (PK) profiles of the chosen drugs in plasma (P), peripheral nerves (N), spinal (S), and brain (B) compartments (theoretical PK curves shown in gray). After the induction of a hyperalgesic state at the left forearm using HFS, the biomarkers will be assessed at four time-points shown in light red: before drug administration, and at three different times after drug administration, close to the expected maximum drug concentration and at relevantly lower drug concentrations. The biomarkers will be derived from measures of LEPs and PEPs elicited by laser and pinprick stimulation of the sensitized and contralateral non-sensitized limb, and measures of the spectral content of ongoing EEG. Patient-reported outcomes will be used to assess subject expectations (expectation PROMs) and state (state PROMs). Hyperalgesia testing (light blue) will be used to assess and compare the HFS-induced hyperalgesia across study periods and across the different BioPain RCTs

As shown in Fig. 1, the biomarkers will be evaluated by assessing the effects of three analgesic or antihyperalgesic agents (lacosamide, pregabalin, tapentadol) and placebo, given as a single dose in four separate study periods separated by at least 1 week.

In each study period, the EEG-derived biomarkers (LEPs and PEPs elicited by stimulation of the left and right forearm, ongoing EEG) will be measured four times: before drug administration, and at three time-points expected to correspond to relevantly different drug concentrations.

Also in each study period, before drug administration and the first biomarker assessment, HFS will be applied to the left forearm in order to induce a sustained state of hyperalgesia restricted to that forearm. This will allow evaluating drug effects on nociceptive processing both for nociceptive input conveyed within normal non-sensitized nociceptive pathways (LEPs and PEPs elicited by stimulation of the non-sensitized forearm) and for nociceptive input conveyed within sensitized nociceptive pathways (LEPs and PEPs elicited by stimulation of the sensitized forearm).

The key variables derived from the EEG measurements that we will evaluate (and which define the primary and key secondary endpoints of the study) are the treatment-induced changes in magnitude of LEPs elicited by laser stimulation of the non-sensitized forearm (peak-to-peak amplitude of the negative-positive vertex potential), the treatment-induced changes in magnitude of PEPs elicited by pinprick stimulation of the sensitized forearm (peak-to-peak amplitude of the negative-positive vertex potential), and the treatment-induced changes in ongoing oscillatory EEG activity (magnitude of theta oscillations).

In addition to these EEG readouts, participants will be asked to rate the intensity and unpleasantness of the percept elicited by the laser and pinprick stimuli used to elicit LEPs and PEPs. These ratings will be used to relate changes in EEG biomarker measurements with subjective changes in pain perception.

Furthermore, as chronic pain is often accompanied by depression and anxiety, assessment tools for these domains will also be applied to the healthy subjects to measure their sensitivity to acute changes, and use them as predictors of individual differences in pain sensitivity. To this end, patient-reported outcome measures (PROMs) will be used to assess subjective pain perception, and validated questionnaires will be used to assess psychological traits and states.

## Methods: participants, interventions, and outcomes

### Study setting {9}

Four clinical sites in four countries will participate: the Department of anesthesiology of the Cliniques universitaires Saint-Luc of the Université catholique de Louvain in Belgium (Principal Investigator: Patricia Lavand’homme), the Center for Biomedicine and Medical Technology Mannheim (CBTM) of the University of Heidelberg in Germany (Principal Investigator: Rolf-Detlef Treede), the Department of Neurology and Psychiatry of the Sapienza University of Rome in Italy (Principal Investigator: Andrea Truini), and the Nuffield Department of Clinical Neurosciences (NDCN) of the University of Oxford in the United Kingdom (Principal Investigator: Irene Tracey). Details on the study sites can be obtained on the EudraCT clinical trials register (2019-001204-37).

All sites are academic hospitals and/or academic laboratories conducting research in human volunteers.

Furthermore, the following participants will assume non-clinical roles in this study:

- Engineering Mathematics and Computing Lab (EMCL), Interdisciplinary Center for Scientific Computing (Heidelberg University), Germany. Contribution: assuming responsibility for data storage and advanced statistical analysis.
- ConsulTech GmbH, Berlin, Germany. Contribution: ConsulTech will coordinate trial monitoring activities. Tasks include review and inspection of the quality of the data and the compliance to and implementation of regulations such as the declaration of Helsinki, GCP, and the Clinical trial plan.
- MRC Systems (spin-off from the University of Heidelberg and the German Cancer Research Center in Heidelberg), Germany. Contribution: MRC Systems will provide to each clinical partner the multipin electrode used to deliver HFS, as well as the mechanical pinprick stimulators for the recording of PEPs and for hyperalgesia testing.
- Pharmacometrics and Systems Pharmacology (PSP), Department of Pharmacy and Pharmaceutical Technology of the School of Pharmacy, University of Navarra, Spain. Contribution: Integrate from a quantitative mechanistic and translational perspective, PK/PD information gathered from the study, as well as PK/PD information provided by preclinical in vitro and in vivo studies conducted within the BioPain subtopic of IMI-PainCare. The end-product will consist of a model formulated on the basis on the known and data-driven mechanisms of action that can be (among several other applications): (i) used through modeling and simulation to optimize dosing scenarios, (ii) applied retrospectively or prospectively in other scenarios to get meaningful PK/PD parameters.
- Grünenthal GmbH, Aachen, Germany. Contribution: co-leading the task to support consensus on final study designs across IMI2-PainCare-BioPain-RCT1 to RCT4. Co-leading the task of clinical study implementation and operations.
- Eli Lilly, research site Erl Wood, UK. Contribution: co-leading the task of data delivery and analysis (preclinical and clinical). Co-leading the task of preclinical biomarker back-translation, including PK.
- ESTEVE, Barcelona, Spain. Contribution: performing bio-analyses of the IMPs as laid down in separate specification manuals.
- Teva Pharmaceutical Industries Ltd., headquartered in Petah Tikva, Israel. Contribution: pharmacometric support, clinical programming, data collection and capturing, and input of expertise related to CDISC.

### Eligibility criteria {10}

There will be an initial screening visit. Following this first screening of inclusion and exclusion criteria, the subject will be either excluded from the trial or scheduled for the first study period. Tables 1 and 2 list the inclusion and exclusion criteria at the screening visit.

**Table 1:** Inclusion criteria at screening visit

	Inclusion criteria at screening visit	Justification / rationale
01	Provision of signed and dated informed consent form	Ethical requirement
02	Stated willingness to comply with all study procedures and regimens and availability for the duration of the study	Ethical requirement and to minimize dropout rate
03	Caucasian male or female subjects, aged 18 to 45 years	To minimize variability. Laser heat stimuli used to elicit LEPs will be delivered to the skin using an Nd:YAP laser. Because skin reflectance, absorption, and transmittance of the infrared radiations generated by this laser are highly dependent on skin pigmentation, only Caucasian participants with light skin will be recruited.
04	Subjects must be in good health as determined by the medical history, physical, and laboratory examinations and must not show any clinically significant deviations from reference ranges as determined by 12-lead electrocardiogram (ECG), vital signs (blood pressure, pulse rate and respiratory rate), and laboratory parameters (renal and hepatic function).	Subject safety and interpretability of results
05	Body mass index > 18 kg/m^2^ and < 30 kg/m^2^ with a minimum body weight of 45.0 kg and a maximum of 100 kg (for men and women)	Consistent with being in good health
06	Ability to take oral medication	Practical reason
07	For female subjects of childbearing potential: use of highly effective contraception with a low failure rate defined as < 1% per year for at least 1 month prior to screening and agreement to use such a method during study participation and for an additional 4 weeks after the end of study drug administration: - Combined (estrogen and progestogen containing) hormonal contraception, - An intra-uterine device (hormone-free), - Progestogen-only hormonal contraception associated with inhibition of ovulation, - An intra-uterine hormone releasing system (IUS) A woman of non-childbearing potential may be included if surgically sterile (i.e., after laparoscopic or hysteroscopic sterilization, hysterectomy or bilateral oophorectomy) or post-menopausal for at least 2 years.	To avoid pregnancies with potential harm to the unborn
08	Right hand dominance (assessed using the Edinburgh Handedness Inventory, and defined as a score ≥ 60)	To minimize variability

**Table 2:** Exclusion criteria at screening visit

	Exclusion criteria at screening visit	Justification / rationale
01	Presence of any medical devices (e.g., cardiac pacemaker), implants, or prothesis unless it is beyond discussion that these will not put the subject’s safety during the study at risk and will not interfere with the results of the study.	To avoid interference with the purpose of the study and to ascertain the subject’s good health
02	Known or suspected allergic reactions or hypersensitivity to components of lacosamide (Vimpat®). Second- or third-degree atrioventricular (AV) block.	Contraindications for lacosamide
03	Known or suspected allergic reactions or hypersensitivity to components of pregabalin (Lyrica®).	Contraindications for pregabalin
04	Known or suspected allergic reactions or hypersensitivity to components of tapentadol (Palexia®). Known contraindication for drugs with μ-opioid agonist activity, i.e., significant respiratory depression, acute or severe bronchial asthma or hypercapnia. Present or suspected paralytic ileus. Acute intoxication with alcohol, hypnotics, centrally acting analgesics, or psychotropic drugs.	Contraindications for tapentadol
05	Not willing or able to abstain from changes in physical exercise activities during the study.	To avoid interference with the purpose of the study
06	Any chronic pain condition or recent (i.e., within the preceding 2 years) history thereof.	To avoid interference with the purpose of the study
07	Migraine (at least 1 attack in the last 24 months).	To avoid interference with the purpose of the study
08	Recurrent headache or back pain on more than 5 days/month in the last 3 months.	To avoid interference with the purpose of the study
09	Caffeine consumption of more than 8 servings of coffee, tea, or other caffeinated drinks per day. Each serving is approximately 120 mg of caffeine.	To avoid interference with the purpose of the study
10	Any relevant symptom of neurological dysfunction of the motor and sensory system that may interfere with the conduct of the study.	To avoid interference with the purpose of the study
11	Clinically evident psychiatric diseases (e.g., depression, anxiety).	To avoid interference with the purpose of the study
12	History or symptoms of central nervous system disease or peripheral nerve lesions or dysfunction with sequelae that may impact the study assessments or that may deteriorate by one dose of a drug with antiepileptic, noradrenergic or opioid activity.	To avoid interference with the purpose of the study Subject safety
13	Focused neurological examination showing signs of abnormality.	To avoid interference with the purpose of the study
14	Active internal disease or sequelae of internal disease (e.g., diabetes mellitus, liver diseases, kidney diseases, cardiovascular diseases, hypo- or hyperthyroidism, hypertension).	To ascertain the subject’s good health
15	Diseases or conditions known to interfere with the distribution, metabolism, or excretion of drugs.	To avoid artifacts
16	Clinically significant disease (e.g., medical history of infection with human immunodeficiency virus (HIV) type 1 or type 2, hepatitis B, or hepatitis C) or condition that may affect efficacy or safety assessments, or any other reasons which, in investigator’s opinion, may preclude the subject’s participation in the trial.	Safety of investigator and their staff Standardization of the trial population
17	Not willing or able to abstain from alcohol from 48 h prior to any study period and until the end of the study period.	To ascertain and protect the subject’s good health and suitability for the study
18	Consumption of cannabis in the last 4 weeks prior to the study.	To ascertain and protect the subject’s good health and suitability for the study
19	Evidence or history of alcohol or drug (opioids, amphetamines, benzodiazepines cannabinoids) abuse (as defined by ICD-10 or DSM IV) including positive or missing drugs of abuse screen (urine drugs of abuse test). Consumption of more than 21 alcohol units per week for male subjects and more than 14 units per week for female subjects (1 alcohol unit = 1 beer [12 oz./355 mL] = 1 wine [5 oz./150 mL] = 1 liquor [1.5 oz./40 mL] = 0.75 oz./20 mL alcohol).	To ascertain and protect the subject’s good health and suitability for the study
20	Habitually smoking more than 10 cigarettes, 2 cigars, or 2 pipes of tobacco per day within the last 6 months before enrollment in this trial.	To ascertain the subject’s good health
21	Known or suspected of not being willing or able to comply with the requirements of the trial protocol or the instructions.	To ascertain the subject’s suitability for the study
22	Inability to communicate meaningfully with the trial site staff (e.g., insufficient language skills).	To ascertain the subject’s safety
23	Any person with direct involvement in the trial conduct; any person under the direct supervision of the investigator or dependent on the investigator.	Ethical requirement
24	Blood loss of 500 mL or more (e.g., owing to blood donation) within 3 months before enrollment in this trial.	To ascertain the subject’s suitability for the study
25	Pregnancy, planned pregnancy or lactation.	Ethical requirement to protect the unborn or newborn child
26	Presence of dermatological conditions in the test areas of the study that would prevent the proper application of study procedures, such as electrodes for HFS, pinprick (dermatitis, psoriasis, contact eczema, local changes of the skin due to regularly playing volleyball etc.).	To avoid interference with the purpose of the study
27	Any other reason to exclude the subject according to judgment by the investigator	To avoid interference with the purpose of the study
A set of *temporary exclusion criteria* have also been defined. The subject will not be excluded if some of these temporary exclusion criteria are met during the screening visit. Instead, the first study period may be postponed. Before the start of the first study period, previously met temporary exclusion criteria will be checked again, and their absence will be verified before the screening for the first study period takes place.		
28	Any drug intake in the past 2 weeks including antibiotics, herbal medicines, and other remedies except the following allowed drugs: oral paracetamol or ibuprofen for a self-limiting condition (e.g., toothache, bruise) for up to 3 days in total within the past 2 weeks; oral antihistaminics and nasal aerosol and topical treatments for seasonal allergy up to 1 week before screening; contraceptives are allowed without time limit.	To ascertain the subject’s good health and to avoid interference with the purpose of the study
29	Any transient illness within 2 weeks before screening.	To ensure the subject’s good health
30	Changes in physical exercise activities, e.g., starting workout/training within 1 week before screening.	To avoid interference with the purpose of the study
31	Current or recent (during the preceding 2 weeks) acute pain lasting more than 4 h.	To avoid interference with the purpose of the study
32	Jet lag / irregular working hours / sleep restriction in the last 3 days before the screening period.	To avoid interference with the purpose of the study

### Who will take informed consent? {26a}

Before any trial-related procedure will be performed, freely given informed consent will be obtained by authorized trial site staff.

The informed consent discussion, the information sheet, and the informed consent form provided to subjects will adhere to GCP and applicable regulatory requirements. The informed consent discussion with the subject will be performed by the Principal Investigator or an appropriately trained delegate. The information sheet and informed consent form will be approved by the relevant IEC(s).

Subjects will be informed as soon as possible if new information becomes available that may be relevant to their willingness to continue participation in the trial. The communication of this information will be documented.

The information sheet and informed consent form will:

- Provide a complete list of all invasive and non-invasive procedures which are part of the study including information on how often these procedures will be performed.
- Describe the discomfort involved in the study.
- Explain that the subject’s participation in the trial is voluntary and that the subject may refuse to participate or withdraw from the trial, at any time, without penalty or loss of benefits to which the subject is otherwise entitled.
- Explain that subject blood samples and data will be shared by the Investigator with non-clinical partners of the trial, naming these partners, specifying the samples and data to be shared, and explicitly obtaining the subjects’ consent for this sharing.
- Detail how unexpected findings resulting from study procedures will be communicated with the subject or the subject’s health care provider, as required by local law and as preferred by the subject. The information sheet and informed consent form will explicitly obtain the subjects’ consent for this policy.
- Describe the collection, storage, and protection of personal data as required by European and national law.
- Explain, if applicable, the possibility that anonymized samples and data could be shared by the Investigator with third parties, naming these parties, specifying the samples and data to be shared, and explicitly obtaining the subjects’ consent for this sharing.

### Additional consent provisions for collection and use of participant data and biological specimens {26b}

The informed consent will explain, if applicable, the possibility that anonymized samples and data could be shared by the Investigator with third parties, naming these parties, specifying the samples and data to be shared, and explicitly obtaining the subjects’ consent for this sharing.

## Interventions

### Explanation for the choice of comparators {6b}

The objective of IMI2-PainCare-BioPain-RCT3 is to test whether parameters derived from non-invasive EEG recordings obtained in healthy volunteers exposed to a single dose of a drug can be used to evaluate the dynamic effects of a given drug on nociceptive signal processing and, thereby, be used as pharmacodynamic biomarkers for analgesic drug development.

Studies to validate the pharmacological concept of new analgesics are commonly conducted in healthy subjects. Therefore, this study is conducted in healthy subjects. In line with the common design of pharmacodynamic studies in healthy subjects, the single-dose, crossover design is adequate for studies of the BioPain project. Randomization, blinding, and placebo control serve to minimize bias.

#### Rationale for the chosen investigational medicinal products (IMPs) and their dose

##### Lacosamide

In the EU, lacosamide has marketing authorization as monotherapy or add-on therapy for epilepsy. In 2008, UCB Pharma withdrew its application for a marketing authorization for lacosamide for the treatment of painful diabetic polyneuropathy. Lacosamide is efficacious in animal models of neuropathic pain [11]. Lacosamide doses of 100 mg, 200 mg, and 300 mg twice daily (BID; i.e., total daily doses of 200 mg, 400 mg, and 600 mg) were studied in placebo-controlled, clinical studies over 10 to 18 weeks in patients with painful diabetic neuropathy [12–15]. In none of the studies did the 100 mg BID dose separate from placebo. In 2 out of 3 studies, statistically significant superiority over placebo was found for the 200 mg BID regimen, although at the cost of a 12.2% rate of premature discontinuations (see lacosamide SmPC in its current valid version). The rate of discontinuations almost doubled from the 200 mg BID to the 300 mg BID regimen and is the most likely cause for the failure of this dose to achieve superiority over placebo. Despite the high rate of discontinuations related to adverse events, no specific safety issue of lacosamide became apparent from these studies as confirmed by the European Withdrawal Assessment Report (EMEA/CHMP/658067/2008) that accompanied the rejection by the EMA of a marketing authorization of lacosamide for neuropathic pain. Recently, a double-blind RCT has shown the effect of lacosamide in patients with nav1.7 mutation-related small fiber neuropathy [16], and a study in painful small fiber neuropathy found that lacosamide normalized the firing pattern of C fibers using microneurography, reduced heat and pain thresholds, and also reverted abnormal excitability of nociceptors derived from human-induced pluripotent stem cells, suggesting a specific modification of the function of peripheral nociceptors [17]. Single oral doses of 200 mg lacosamide (2 film-coated tablets of 100 mg each simultaneously) have reportedly been administered to 67 healthy, male, Caucasian subjects [18, 19]. Three out of these 67 subjects discontinued prematurely from the study because of adverse events which were judged not to be medication related (epiglottitis, common cold). In these studies, a total of 40 adverse events were observed after oral administration of lacosamide. The most commonly observed adverse events were dizziness, tiredness, fatigue, paresthesia surrounding the mouth, and thrombophlebitis. In conclusion, there is adequate evidence that a 200 mg BID dose of lacosamide, but not a lower dose, has a relevant analgesic effect. A single dose of 200 mg lacosamide is an acceptable single dose for a biomarker study in healthy subjects.

##### Pregabalin

In the EU, pregabalin has marketing authorization for the treatment of peripheral and central neuropathic pain in adults. The dose range is 150 to 600 mg per day given in either two or three divided doses. Single oral doses of 300 mg pregabalin have been administered to almost 200 healthy subjects [20–23]. The intensity of AEs ranged from mild to severe. Most frequently occurring AEs were dizziness, somnolence, fatigue, and euphoric mood. No subject was withdrawn from the study for safety reasons. There is adequate evidence that a 150-mg dose of pregabalin has a clinically relevant analgesic effect in contrast to 75 mg daily which is not effective. In conclusion, a single dose of 150 mg pregabalin seems an acceptable single dose for a biomarker study in healthy subjects.

##### Tapentadol

In the EU, tapentadol has marketing authorization for the treatment of peripheral and central neuropathic pain in adults. The recommended oral starting dose of the immediate-release tablets is 50, 75, or 100 mg depending on pain intensity. Single oral doses, up to 250 mg, have been given to healthy subjects. However, in the context of a thorough QT study, oral doses of 100 mg tapentadol administered to healthy subjects are referred to as highest therapeutic doses and 150 mg as supratherapeutic doses. In conclusion, a 100-mg dose seems a justifiable single dose for a biomarker study in healthy subjects.

#### Rationale for the induction of hyperalgesia using high-frequency stimulation (HFS)

The effects of the IMPs on nociceptive processing will be assessed concurrently in both a non-sensitized normal condition and a clinically relevant hyperalgesic condition. High-frequency electrical pulses delivered to the skin using a multipin electrode designed to preferentially activate cutaneous nociceptors [9] is a validated and non-invasive procedure to induce this hyperalgesic condition. Numerous studies have shown that cutaneous HFS delivered using this electrode (e.g., 100 Hz trains lasting 1 s of 2-ms electrical pulses, repeated 5 times at an intensity sufficient to generate strong activity in small-diameter nociceptive afferents) leads to a stable hyperalgesia [9, 10] lasting at least 4 h [24]. Cutaneous HFS generates a marked secondary hyperalgesia due to central sensitization, primarily manifesting itself by an increased sensitivity to mechanical pinprick stimulation. Importantly, HFS does not generate any confounding long-lasting spontaneous after sensation. Cutaneous HFS does, however, induce a local skin flare response, indicative of neurogenic inflammation that can be exploited to monitor peripheral sensitization effects [9].

### Intervention description {11a}

The IMPs will be (1) lacosamide (Vimpat®) film-coated tablets (composition: 2 × 100 mg lacosamide tablets); (2) pregabalin (Lyrica®) capsules (composition: 2 × 75 mg pregabalin capsules); (3) tapentadol (Palexia®) immediate-release tablets (composition: 2 × 50 mg tapentadol immediate-release tablet); and placebo capsules (composition: 2× hard gelatine capsules filled with mannitol and colloidal silicon dioxide).

Each IMP will be administered overencapsulated as single oral dose (two capsules), in a double-blind, 4-period, crossover fashion where the study periods are separated by at least 1 week. All IMPs, except placebo, are registered medications in the countries that will participate in the trial. IMPs will be obtained from commercial stock.

For the induction of hyperalgesia, HFS will be delivered to superficial nerve terminals using a multipin surface electrode similar to the electrode used in Klein et al. [9] and developed by MRC. The stimuli will be applied to the skin of the left volar forearm. The electrical pulses will be generated by a standard, CE-approved, constant-current electrical stimulator routinely used for clinical diagnostic purposes. The stimulation will consist in trains of 100 Hz pulses lasting 1 s and repeated five times, at an intensity sufficient to generate strong activity in small-diameter nociceptive afferents.

### Criteria for discontinuing or modifying allocated interventions {11b}

At the start of each of the four study periods, subjects will be excluded from the period if any of the criteria listed in Table 3 apply.

**Table 3:** Exclusion criteria at study periods

	Exclusion criteria at study periods:	Justification / rationale
33	For female subjects of child bearing potential: positive or missing pregnancy test	To protect a fetus
34	Positive or missing urine test for drugs of abuse (opioids, amphetamines, benzodiazepines, cannabinoids).	Subject safety and to avoid interactions with, e.g., tapentadol (PD interactions, safety interactions)
35	Blood loss of 500 mL or more (e.g., owing to blood donation) since screening.	To ascertain the subject’s suitability for the study
36	Any other reason to exclude the subject according to judgment by the investigator	To avoid interference with the purpose of the study.
Temporary exclusion criteria at study periods. The subject is not excluded if some of these temporary exclusion criteria are met at screening of the study period. Instead, the study period may be postponed. If this is the case, all temporary exclusion criteria will be checked again.		
37	Alcohol consumption in the last 48 h prior to the study period.	Subject safety and to avoid interactions with, e.g., tapentadol (PD interactions, safety interactions)
38	Intake of any drug including herbal medicines and other remedies except the following: contraceptives; oral paracetamol or ibuprofen up to the maximum recommended dose according SmPC, with last intake for both > 4 days prior to each study period for a self-resolving condition.	As described for screening visit
39	Changes in physical exercise activities, e.g., starting workout/training within 1 week prior to the study.	To avoid interference with the purpose of the study
40	Current pain within the last 4 days before the study period.	To avoid interference with the purpose of the study
41	Any transient, clinically relevant illness within 4 days before the period.	To ensure the subject’s good health
42	Incidentally not willing or able to comply with the requirements of the trial protocol or the instructions or to communicate meaningfully with the trial site staff.	To ascertain the subject’s suitability for the study
43	Incidentally unable to take oral medication.	Requirement for the study
44	Jet lag / irregular working hours / sleep restriction in the last 3 days before the period.	To avoid interference with the purpose of the study

### Strategies to improve adherence to interventions {11c}

At each study period, an oral dose of lacosamide, pregabalin, tapentadol, or placebo will be taken with 100 mL of plain water. After intake of the study medication, the investigator will inspect the subject’s mouth to verify that the medication has been swallowed.

### Relevant concomitant care permitted or prohibited during the trial {11d}

At screening, any drug intake in the past 2 weeks including antibiotics, herbal medicines, and other remedies are prohibited except the following allowed drugs: oral paracetamol or ibuprofen for a self-limiting condition (e.g., toothache, bruise) for up to 3 days in total within the past 2 weeks; oral antihistaminic and nasal aerosol and topical treatments for seasonal allergy up to 1 week before screening; contraceptives are allowed without time limit (see exclusion criterion #28). At the beginning of each of the four study periods, intake of any drug including herbal medicines and other remedies are prohibited except the following: contraceptives; oral paracetamol or ibuprofen up to the maximum recommended dose according SmPC, with last intake for both > 4 days prior to each study period for a self-resolving condition (see exclusion criterion #38). If these temporary exclusion criteria are met, the study period may be postponed.

### Provisions for post-trial care {30}

IMI2-PainCare-BioPain-RCT3 is a study conducted in healthy volunteers. No post-trial care is thus foreseen. Between 7 and 14 days after the end of the last study period, the absence of untoward medical or mental sequelae of the study will be ascertained in a follow-up telephone call with the subject.

At each participating site, the Principal Investigator will arrange suitable insurance for the subjects included in this trial. The Principal Investigator will inform all subjects about this insurance and (if requested) be prepared to explain the relevant terms and conditions of this insurance to the subject.

If changes to the trial are implemented after the initial insurance was arranged, e.g., due to protocol amendments, the Principal Investigator will notify the insurance company of these changes in accordance with the insurance conditions. If changes to insurance arise, the Principal Investigator will inform the subjects at his trial site about relevant changes.

### Outcomes {12}

The objective of the study is to evaluate a set of biomarkers derived from non-invasive EEG recordings that could be used to evaluate target engagement of drugs acting on nociceptive processing. The effects of the IMPs on nociceptive processing will be assessed by recording LEPs, PEPs, and ongoing EEG.

#### Rationale for the chosen biomarkers

LEPs are event-related brain potentials related to the activation of rapidly adapting heat-sensitive polymodal A-fiber nociceptors (Type 2 A-fiber mechano- and heat-sensitive fibers, Type2-AMH) [25]. Their magnitude is thus dependent on the activation of these nociceptive afferents, the transmission of nociceptive input within these afferents, and the further transmission and processing of that input within the spinothalamic system. Studies have shown that their magnitude can be modulated by drugs exerting an effect on the nociceptive system. For example, Petersen-Felix et al. [26] showed that IV infusion of alfentanil significantly reduces the magnitude of the N2-P2 component of LEPs. Similarly, Truini et al. [27] showed an average N2 and P2 amplitude reduction of 50% 60 min after IM injection of Tramadol (100 mg).

PEPs are event-related brain potentials related to the activation of mechano-sensitive A-fiber nociceptors [4]. Their magnitude is thus dependent on the activation of these mechano-sensitive afferents, the transmission of nociceptive input within these afferents, and most importantly, their further transmission and processing within the spinothalamic system. Most importantly, studies have shown that, after inducing central sensitization using HFS, the magnitude of PEPs elicited by stimuli delivered to the area of secondary hyperalgesia is enhanced [5], indicating that changes in PEP magnitude can be used as an index of central sensitization.

The magnitude of ongoing EEG oscillations has been shown to be sensitive to changes in brain network function. Several studies have shown that modulation of the magnitude of ongoing theta band oscillations (4–7 Hz) can relate to perceived pain intensity [6, 28], but also to variations in vigilance and attentional states [29, 30]. Other studies have shown that many drugs acting on the central nervous system can modulate the magnitude of ongoing EEG oscillations [7, 31].

#### Primary and key secondary outcomes

The two primary and three key secondary endpoints (Table 4) are the changes in the magnitude of pain-evoked EEG responses (LEPs: peak-to-peak amplitude of the negative-positive vertex potential elicited by laser stimulation of the non-sensitized forearm; PEPs: peak-to-peak amplitude of the negative-positive vertex potential elicited by pinprick stimulation of the sensitized forearm) and ongoing oscillatory EEG activity (magnitude of theta oscillations).

**Table 4:** Primary and key secondary endpoints

**Primary endpoints:**	
1. To test if the percentage reduction of LEP amplitude 60 min post-drug administration differs in the tapentadol period as compared to the placebo period, at the non-sensitized forearm.	
2. To test if the percentage reduction of PEP amplitude 60 min post-drug administration differs in the tapentadol period as compared to the placebo period, at the sensitized forearm.	
**Key secondary analyses of the primary endpoints:**	
1. To test if the percentage reduction of LEP amplitude 60 min post-drug administration differs in the pregabalin and/or lacosamide periods as compared to the placebo period, at the non-sensitized forearm.	
2. To test if the percentage reduction of PEP amplitude 60 min post-drug administration differs in the pregabalin and/or lacosamide periods as compared to the placebo period, at the sensitized forearm.	
3. To test if the percentage change in magnitude of theta oscillations 60 min post-drug administration differs in the tapentadol, pregabalin, and/or lacosamide periods as compared to the placebo period.	

### Participant timeline {13}

**Fig. 2:**
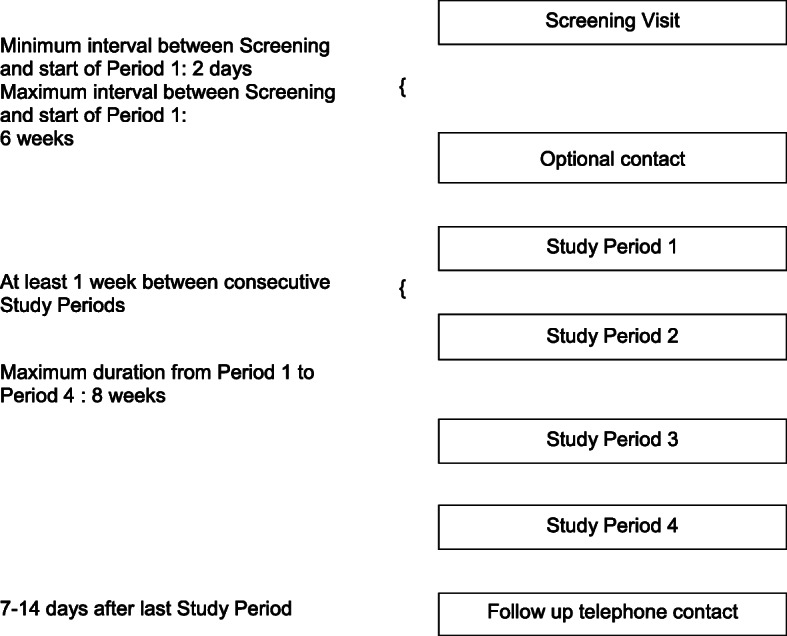
Timeline of the study which includes a screening visit, an optional contact, four study periods separated by at least 1 week, and a follow-up telephone contact

Figure 2 summarizes the participant timeline which includes a screening visit, followed by four study periods and a follow-up telephone contact. There will be an optional contact before the first study period. Following screening of inclusion and exclusion criteria, the subject is either excluded from the trial or scheduled for study period 1. If screening shows that one or more temporary exclusion criteria are met, the subject can be included. However, in this case, before the start of study period 1, the previously met temporary exclusion criteria of the screening visit will be checked again, and their absence will be verified before period 1 takes place. The maximum time between the screening visit and the start of study period 1 is 6 weeks.

If screening of exclusion criteria for eligibility for period 1 shows that one or more temporary exclusion criteria are met, the start of period 1 can be postponed and re-scheduled. In this case, the previously met temporary exclusion criteria of the study period will be checked again and their absence will be verified before the subject is eligible for the period. This procedure may be repeated for individual periods 1 to 4.

The four study periods are separated by washout periods of at least 1 week. The maximum time between study period 1 and 4 is 8 weeks. Between 7 and 14 days after the last study period, the absence of untoward medical sequelae of the study will be ascertained in a follow-up telephone call with the subject.

Each subject is expected to be in the trial for approximately a minimum of 30 days and a maximum of 14 weeks.

#### Screening visit

The following will be performed:

- Explain the purpose of the research, the extent and burden of the procedures and assessments.
- Obtain informed consent.
- Assess subject handedness using the Edinburgh Handedness Inventory.
- Record demographic data.
- Record prior and concomitant medication.
- Record clinically relevant medical and surgical history.
- Assess inclusion criteria.
- Perform a focused neurological examination in the presence of any clinically evident sensory disorder and a physical examination if indicated from the medical history.
- Record a 12-lead electrocardiogram and verify absence of signs of second- or third-degree atrioventricular block.
- Perform urine pregnancy test.
- Collect a blood sample to verify normal renal and hepatic functions.
- Perform urine test for drug abuse (opioids, amphetamines, cannabinoids) and perform alcohol consumption check.
- Record psychosocial characteristics using patient-reported outcome measures and validated questionnaires.
- Instruct the subject on the study-specific procedures including how to use the rating scales.
- Demonstrate the test stimuli that will be used, induce sensitization at the left forearm using HFS, and perform hyperalgesia testing 20 min after induction.
- Assess exclusion criteria specific for the screening visit.

#### Optional contact before start of treatment period

The optional (telephone / e-mail) contact will be after the screening visit and at the latest 48 h before the first study period. The following will be performed:

- Arrange for the subject to attend the first study period. Arrangement for the subsequent periods may be made at the same time or at the end of each study period.
- Remind the subject to abstain from alcohol during 48 h preceding each study period.
- Ascertain that the subject has not taken any drugs in the 4 days preceding each study period—except contraceptives—and/or did not suffer from transient illness.
- Remind the subject that breakfast should be taken before arrival at the site.

#### Treatment periods: study periods 1, 2, 3, and 4

Each study period will be separated by at least 1 week. The subjects will have a light breakfast at home. The schedule of events is identical for all study periods and is provided in Table 5. Drinks (water or sugared juice, e.g., apple juice) and a light meal will be served as scheduled in Table 5. The time relative to drug administration is the leading time. The clock times are indicative only and may vary from site to site. Times of assessments and procedures should be adhered to as closely as possible. However, precise recording of exact times of assessments is more important than strict adherence to times.

**Table 5:**
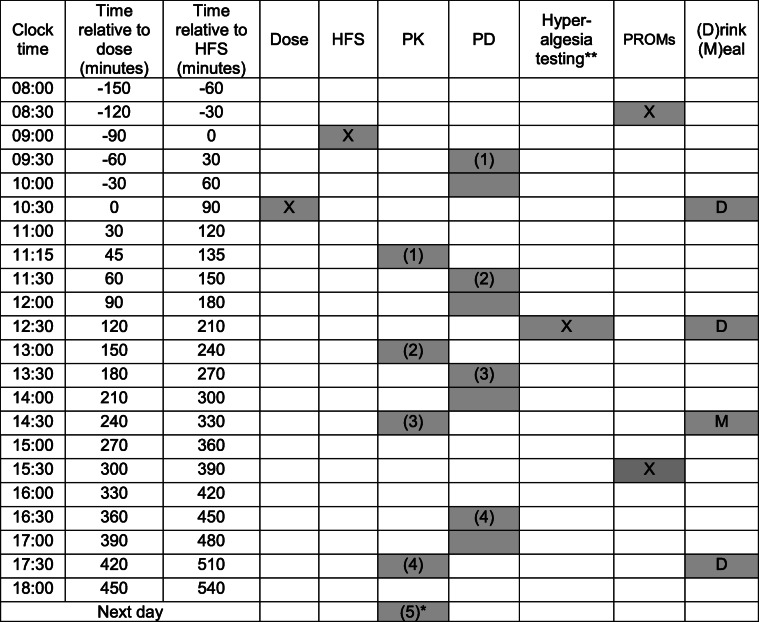
Detailed timetable of procedures and assessments in periods 1, 2, 3, and 4

* The PK sample on next day can be taken at any suitable time provided that the exact time of sampling is precisely recorded. ** Hyperalgesia testing at the sensitized and contralateral forearm, harmonized across all four IMI2-PainCare-BioPain RCTs

Procedures, assessments, and events during a period:

- The subject will have breakfast at home and arrive at the site at or before 08:00 AM.
- Record prior and concomitant medication.
- Urine screening test for drugs of abuse and alcohol consumption check.
- For female subjects: urine pregnancy test.
- Reassess subject eligibility for the study according to inclusion and exclusion criteria.
- Train / instruct again the subject on the study-specific procedures.
- Complete PROMs assessing subject expectations.
- Optionally, according to local practices, an indwelling venous catheter will be inserted at the start of each study period and will be left in place for the duration of the study day.
- HFS will be applied to the left forearm to induce sensitization.
- IMP administration.
- A total of 5 blood samples (6 mL each) will be taken as scheduled in Table 5 for pharmacokinetic (PK) analyses. The last sample will be taken on the next day, at any suitable time.
- One pre-dose and 3 post-dose pharmacodynamic (PD) biomarker assessments will be made as scheduled in Table 5.
- Hyperalgesia testing at the sensitized and contralateral forearm will be made as scheduled in Table 5, harmonized across all four IMI2-PainCare-BioPain-RCTs.
- Complete PROMs assessing tiredness and anxiety.
- Drinks (water or sugared juice, e.g., apple juice) and a light meal will be served as scheduled in Table 5.
- Instruct the subject not to drive or bike or operate machinery for the entire day (risk of sedation or dizziness caused by IMP). Instruct the participants that they should not drive or bike or operate machinery on the following day if they feel drowsy or dizzy.
- Upon leaving the trial site, if the subject is feeling drowsy or dizzy, arrange for the participant to be driven home by taxi.

#### Follow-up telephone call

Between 7 and 14 days after the end of the last study period, the absence of untoward medical or mental sequelae of the study will be ascertained in a follow-up telephone call with the subject.

### Sample size {14}

Given the rationale of improving preclinical to clinical translation of pain effects and the intention to use these biomarkers in future early clinical pharmacology studies, a robust signal in a small number of subjects is required if this biomarker is to prove useful in early drug development. In fact, if the investigated drugs and measured effects do not show robust (i.e., significant primary) biomarker signals on the basis of several tens of subjects, the overall usefulness of this biomarker is rather limited. Therefore, the following illustration of the estimated power of this study with a range of 10 to 30 subjects (in total) should serve as justification for the (evaluable) sample size to be chosen at 12 overall (preferably at one center with additional centers providing data for reliability testing at similar sample sizes).

**Fig. 3:**
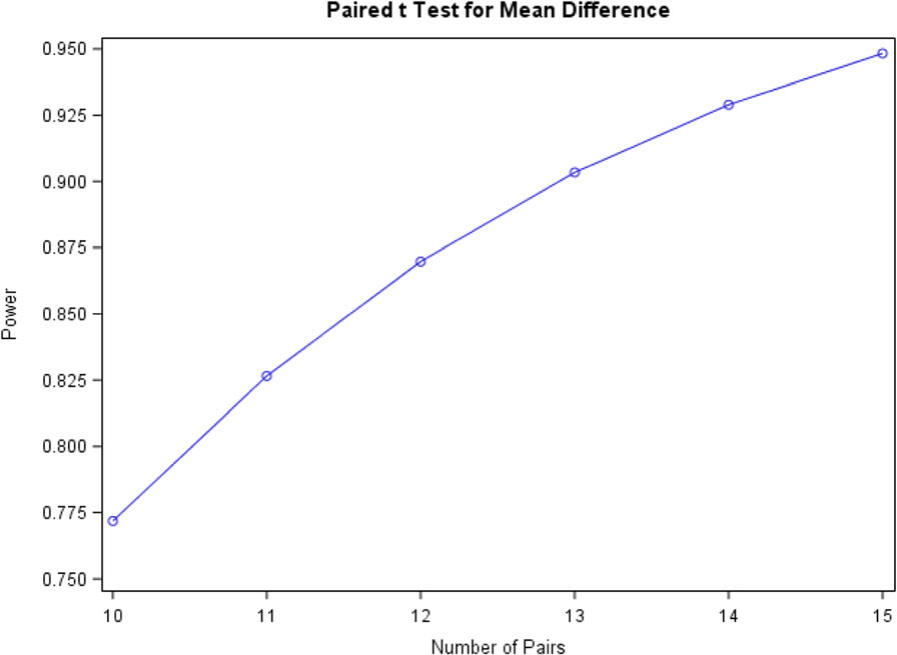
Power as a function of number of pairs (paired *t* test for mean difference)

Knowledge on the variability and effect sizes of EEG parameters are available with tramadol versus placebo as treatment in healthy subjects [27], with alfentanil versus baseline in healthy subjects [26], as well as before versus after morphine treatment in chronic pain patients [32]. The study by Truini et al. [27] conveys effects (i.e., group differences of the LEP N2-P2 changes relative to baseline) around 60% (placebo group) and 90% (tramadol group), i.e., a 30% group difference. The provided data by Petersen-Felix et al. [26] suggests standard deviation (SD) parameters for LEP peak-to-peak amplitude of the negative-positive vertex to be around 25%. With the tapentadol versus placebo comparison of the primary analysis (on LEP peak-to-peak amplitude relative changes from baseline, 2-sided type I error of *α*/2 = 0.025) being essentially a paired *t* test with assumed correlation of 0.3, the power graph shown in Fig. 3 yields 12 evaluable subjects in total to be a reasonable sample size for at least 85% power. While this appears sufficient for the primary analysis, data for similar numbers of subjects would be recommended from all centers for test-retest reliability investigations. Therefore, sample sizes of 8 to 12 subjects treated for all four study periods are required in each participating center.

A potentially high dropout rate and missing data needs to be taken into consideration given the complexity of the design and procedures. This is already addressed by the analysis approach, but to compensate for potentially 20% dropouts, a total of 12–16 subjects will be enrolled per participating center (in the sense of randomizing subjects to enter the treatment phase).

As mentioned above, in case significant signals are not found in this study as conclusion, this is already valuable information for the broader objective of the trial. It should also be noted that the insights into the characteristics of the investigated drugs and biomarkers (cf. PK/PD analyses and/or test-retest reliability) also contribute to the trial rationale irrespective of any significant finding in the primary analysis.

### Recruitment {15}

Healthy subjects will be recruited by advertisements on webpages, newspapers, university billboards, etc. The recruitment rate is anticipated to be 3–4/month per center. Subjects will be pre-screened to identify subjects who could potentially be enrolled into the trial.

#### Screen failures

Screen failures are defined as subjects who consent to participate in the clinical trial but are not subsequently randomly assigned to the IMP. A minimal set of screen failure information is required to ensure transparent reporting of screen failure subjects, to meet the Consolidated Standards of Reporting Trials (CONSORT) publishing requirements and to respond to queries from regulatory authorities. Minimal information includes demography, screen failure details, eligibility criteria, and any serious adverse event (SAE).

#### Subject discontinuation from the trial

Once a subject enrolls in the trial, whilst protecting subject safety, the trial site will make every effort to retain the subject for the planned duration of the trial. A subject may withdraw consent at any time. This will automatically lead to discontinuation of the subject from the trial. The investigator will discontinue the subject’s participation in the study if further participation would involve unjustifiable risk to the subject’s mental or physical well-being or if participation would be against the purpose and interest of the study.

In general, subjects who discontinue are those who complete the end of trial earlier than the end of study period 4. Subjects who discontinue their participation in the trial will not be replaced. The Principal Investigator will document on the case report form (CRF) any discontinuation of a subject and inform the sponsor. Where applicable, the relevant IEC(s) must be informed with a detailed written explanation.

The following will be done for all discontinued subjects, including those who withdrew informed consent:

- Document the main reason for discontinuation from the trial.
- Ensure that all data collected until the time of discontinuation is transferred to the CRF.
- Complete any other trial-related formalities, e.g., those related to discharge from the trial site.
- For subjects withdrawing consent, document in the source data the date and time of withdrawal.
- If a subject withdraws consent and agrees (documented in writing) conduct the follow-up telephone call.

### Assignment of interventions: allocation

#### Sequence generation {16a}

All four study periods of the trial will be double-blinded.

Lacosamide, pregabalin, tapentadol or placebo will be assigned to each subject by a double-blind randomization schedule.

Randomization will be by site. At the first study period day before first IMP administration, subjects will be randomized to receive the lowest available randomization number at the site.

The randomization list (per site) will be built in blocks of four “4-period sequences,” these sequences being random permutations of the four 4-period sequences of a (basic) Latin square. Each block (allowing the sequential allocation of four participants) will use an own (new) random permutation of the Latin squares’ four rows.

The basic Latin square will be chosen randomly from a selection of the 24 available, cf. Table 5.1 in Senn [33], namely the Williams squares therein, with the constraint that each medication precedes each other medication once in this Latin square.

If two (or more) basic Latin squares are used, e.g., permuted rows of the first for randomization of the initial four participants and the permuted rows of the second for the subsequent four participants, then the choice of Latin square per block will also be made randomly.

#### Concealment mechanism {16b}

Each IMP will be administered overencapsulated as single oral dose (two capsules).

A sealed decoding envelope per treatment period will be provided for each randomization number. Each envelope will contain the identification of the IMP allocated to that subject.

#### Implementation {16c}

The IMPs lacosamide (Vimpat®), tapentadol (Palexia®), and pregabalin (Lyrica®) will be purchased centrally by the Heidelberg University Hospital Pharmacy. The pharmacy will perform the manufacturing of placebo capsules and the overencapsulation of the IMPs Lyrica, Palexia, and Vimpat. The IMP will be packaged and labeled centrally by the pharmacy and will be in compliance with applicable local regulations. Detailed information about the packaging and labeling is laid down in a specification document (available on request) with written instructions on, for example, processing, labeling, packaging, quality control, release, storage, and/or delivery of the IMP. The allocation sequence and sealed envelopes will be generated by the Heidelberg University Hospital Pharmacy.

Storage conditions will be specified on the labels of the IMPs according to applicable EU and local regulation. The IMPs will be stored in a secure place with restricted access and temperature monitoring. The IMP delivery (shipping), return, and disposal will be performed in accordance with the specification document (available on request). Controls will be implemented at the trial site to ensure documented compliance with these requirements.

As tapentadol is one of the IMPs in this study, all sites need to be licensed according to local law for the receipt, storage, handling, and administration of narcotics.

General unblinding will only take place after the trial has been completed and the database is locked. Except for single-subject unblinding in emergency situations, investigator unblinding will only be carried out after database lock. Unblinding will be performed by the statistician.

## Assignment of interventions: blinding

### Who will be blinded {17a}

The investigator/trial personnel and subjects will be blinded to the assignment of pregabalin, tapentadol, lacosamide, and placebo (double-blind procedure).

The personnel analyzing the plasma samples for PK analysis will be unblinded during the bioanalytical analysis, but will supply their data to the trial database in a blinded fashion.

### Procedure for unblinding if needed {17b}

The investigator will receive appropriate methods for single case unblinding (i.e., sealed decoding envelopes per treatment period for each randomization number which contain the individual treatment assignment).

Each envelope contains the identification of the IMP allocated to that subject. The investigator may only break the code (open the envelope) when it is necessary and in the subject’s interest to identify the IMP given (e.g., if knowing the identification of the treatment would lead to the investigator treating the subject differently).

For every subject whose blinding code was broken, the following information will be documented:

- The reason for, the date, and time of unblinding.
- The person performing the unblinding.
- The persons informed of the treatment allocation/randomization.

In order to maintain the double-blind nature of the trial, the allocated treatment for a subject will not be communicated further unless required for the surveillance of the subject or, if necessary, for urgent risk to benefit re-evaluation and/or measures for urgent risk minimization. If required by local regulations, it may be that the IEC needs to be informed.

## Data collection and management

### Plans for assessment and collection of outcomes {18a}

#### Collection of pharmacokinetic data

Venous blood samples will be collected at the scheduled time-points (detailed in Table 5) after drug administration and will be used to determine drug plasma concentrations to be used for PK modeling and subsequent PK/PD analysis.

An overview of the total amount of blood to be collected from each study participant is provided in Table 6. In each study period, 5 blood samples, 6 mL each, will be drawn into tubes containing K2-EDTA as anticoagulant and will be centrifuged within 30 min after collection. The harvested plasma will be frozen at − 20 to − 80 °C within 1 h of sampling and kept frozen until sent for analysis. Detailed descriptions of the sample procurement and processing will be provided in a separate specification manual. Bioanalysis will be performed by ESTEVE using validated methods.

**Table 6:** Overview of blood sampling volumes during the study

	Volume / sample (mL)	Number of samples	Total volume (mL)
Screening visit	6	1	6
Optional visit	6	1	6
Pharmacokinetics:			
Period 1	6	5	30
Period 2	6	5	30
Period 3	6	5	30
Period 4	6	5	30
Maximum total volume of blood per subject (mL):			132

#### Collection of demographic data and other baseline characteristics

##### Demographic data

Demographic data to be collected and recorded for this trial are age, sex, body weight, and height.

##### Prior and concomitant medication

All medication requiring prescriptions (including oral contraceptives) and/or over-the-counter medication used within 10 days prior to enrollment and up to the end of the trial will be recorded. Any change in dosage, regimen, or route, during the study will be recorded as a new entry.

##### Relevant prior/concomitant disease or surgical interventions

Current medical conditions and conditions within the last 7 days and controlled by medication will be recorded. Childhood illnesses do not need to be documented, unless they are considered to potentially affect the assessment of the biomarker or could potentially give rise to an adverse event based on the pharmacology of the IMP or related to trial procedures.

##### Physical examination

At the screening visit, a focused neurological examination will be performed in the presence of any clinically evident sensory disorder.

##### Electrocardiography

At screening, a 12-lead electrocardiogram (ECG) will be recorded in supine position after at least 10 min rest. Electrodes will be positioned in accordance with the recommendations of the American Heart Association: three cycles 12-lead ECG and simultaneous rhythm strip at 25 mm/s with a gain setting of 10 mm/mV. The ECG will specifically be checked for signs of 2nd or 3rd degree atrioventricular (AV) block (exclusion criterion, contraindication for lacosamide).

##### Urine pregnancy test

At screening and at the beginning of each study period, a urine pregnancy test will be performed using suitably validated methods commonly used at the site.

##### Alcohol consumption check

At screening and at the beginning of each period, abstinence of consumption of alcohol will be checked.

##### Urine drugs of abuse test

At screening and at the beginning of each period, urine samples provided by the subject will be tested for the presence of opiates, amphetamines, and cannabinoids using suitable validated methods commonly used at the site. This is to avoid that tapentadol (tapentadol has μ-opioid activity) is administered to subjects who have recently taken substances with relevant CNS depressant activity as this may increase the risk of sedation (safety risk if the subject leaves the site, negative impact on study results), dizziness (safety risk), and respiratory depression (safety risk).

##### Laboratory parameters

At screening, a blood sample will be collected to verify normal renal and hepatic functions, following the recommendations of Breithaupt-Groegler et al. (2017) for trials with clinically established IMPs. Renal function will be assessed using the Glomerular Filtration Rate (GFR, estimated by the MDRD formulae (Modification of Diet in Renal Disease – Annals of Internal Medicine 1999;130: 461–470). Normal GFR is defined as ≥ 60 mL/min/1.73 m^2^. Hepatic function will be assessed by measuring alanine aminotransferase (ALT), aspartate aminotransferase (AST), gamma-glutamyltransferase (GGT), and total bilirubin. Values will be considered as acceptable if they are < 1.1× the upper normal threshold as established by the local laboratory performing the analysis. Laboratory results remain valid if they have been completed within 21 days before the first study period. In other case, a new blood sample collection and analysis will be performed during an optional visit (Fig. 2).

#### Collection of pharmacodynamic data

All participating centers have the equipment and expertise required for acquisition of the pharmacodynamic data. Signal processing of the raw PD data to extract the parameters of interest will be done centrally at UCLouvain using the Letswave 6 Matlab toolbox for the analysis of EEG data (http://letswave.org; UCLouvain).

##### EEG/ERP-derived pharmacodynamic data

**Fig. 4:**
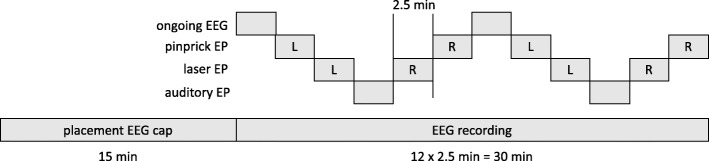
Time course of each biomarker assessment time-point, during which ongoing EEG, pinprick-evoked potentials elicited by stimulation of the left and right forearm, laser-evoked potentials elicited by stimulation of the left and right forearm, and auditory-evoked potentials will be recorded twice (L: left, R: right)

The EEG will be recorded continuously during each biomarker assessment time-point, using a multichannel EEG amplifier and digitizer as detailed in a separate operational manual. Setting-up the EEG recording system will require 10–20 min. Actual collection of the pharmacodynamic data will require approximately 30 min. During the entire assessment, subjects will be seated in a comfortable chair. The assessment consists in the serial acquisition of (1) ongoing EEG at rest, (2) PEPs elicited by stimulation of the left forearm, (3) LEPs elicited by stimulation of the left forearm, (4) auditory-evoked potentials (AEPs), (5) LEPs elicited by stimulation of the right forearm, and (6) PEPs elicited by stimulation of the right forearm. This serial acquisition is repeated twice. Each acquisition block lasts approximately 2.5 min. The time course of the assessment is shown in Fig. 4.

From the collected EEG data, the following biomarkers will be extracted:

- Ongoing EEG. Two 2.5 min recording of ongoing EEG will be performed, while the participant is at rest, and not exposed to any sensory stimuli. Each recording will include 1 min of EEG recording with eyes opened, one 30-s period with eyes closed, followed by a second 1-min recording with eyes opened. A frequency analysis of the magnitude of ongoing EEG oscillations will be used to assess the amplitude of ongoing EEG oscillations within the delta (1–4 Hz), theta (4–7 Hz), alpha (8–12 Hz), beta (15–30 Hz), and gamma (40–90 Hz) oscillations, eyes opened and eyes closed. The signal processing steps are described in the operational manual. This will allow generating the key secondary endpoint “theta_amp” by averaging the amplitude of theta band oscillations across the two 1-min EEG segments eyes opened.
- Laser-evoked brain potentials (LEP). Brief laser heat stimuli lasting only a few milliseconds will be applied to the skin to activate selectively heat-sensitive nociceptors of the skin, as detailed in the operational manual. The thermal stimuli will be produced by a commercially available infrared neodynium:yttrium aluminum perovskite (Nd:YAP) laser stimulator designed for the recording of LEPs. Intensity of stimulation will be set to consistently activate A-delta and C fiber heat-sensitive nociceptors. The device will be used in accordance with safety guidelines, by trained and authorized personnel, as detailed in the operational manual. The Nd:YAP laser is routinely used for diagnostic purposes [34, 35]. The laser stimulator generates a radiant heat stimulus able to heat the skin above the thermal activation of heat-sensitive nociceptors in only a few milliseconds, without producing any skin lesion. The sensations produced by laser stimuli are often described as a brief pinprick, a light burn, or a diffuse warmth. They may be perceived as unpleasant. On rare occasions, the laser stimulus produces a slight punctuate erythema, which may subsequently become hyperpigmented, but always vanishes completely within a few days [36]. In each of the four LEP recording blocks (two blocks per forearm), the laser stimuli will be repeated 15 times, with a randomly varying inter-stimulus interval. The target of the laser stimulus will be slightly displaced during each inter-stimulus interval, to avoid overheating the skin and/or inducing receptor sensitization and/or habituation. After each laser stimulus, the subject will be requested to rate the intensity of the elicited sensation as detailed in the next section. After each stimulation block, the subject will be requested to rate the unpleasantness of the elicited sensation, as detailed in the next section. This EEG data will allow measuring the average amplitude of the negative-positive (N2-P2) vertex potential elicited by laser stimulation [3], required to generate the primary endpoint “LEP N2-P2_amp,” also used for the key secondary analyses. The processing steps to obtain this measurement are detailed in a separate operational manual.
- Pinprick-evoked brain potentials (PEP). Preferential activation of mechano-sensitive skin nociceptors will be achieved by applying calibrated mechanical pinprick stimuli using small surface probes. The device, provided by MRC Systems, is detailed in a separate operational manual. The time of contact of the needle with the skin is obtained using an internal switch which generates a trigger marking the onset of each stimulus in the ongoing EEG recording. The stimuli will be applied manually by the operator, as detailed in the operational manual. The sensation generated by pinprick stimulation is most often described as pricking. The probe does not penetrate the skin and, hence, does not produce any lesion. For each of the four PEP recording blocks (left forearm: two blocks, right forearm: two blocks), the pinprick stimuli will be repeated 15 times, with a randomly varying inter-stimulus interval. The target of the pinprick stimulus will be slightly displaced during each inter-stimulus interval, to avoid receptor sensitization and/or habituation. After each pinprick stimulus, the subject will be requested to rate the intensity of the elicited sensation. After each stimulation block, the subject will be requested to rate the unpleasantness of the elicited sensation. This EEG data will allow measuring the average amplitude of the negative-positive (N2-P2) complex elicited by pinprick stimulation [5], required to generate the primary endpoint “PEP N2-P2_amp,” also used for the key secondary analyses. The processing steps to obtain this measurement are detailed in a separate operational manual.
- Auditory-evoked brain potentials (AEP). In addition to exerting a selective effect on nociception, the medications as well as the procedure used to induce hyperalgesia may induce unspecific changes in brain function, such as changes in the level of vigilance (e.g., drug-induced sedation). For this reason, AEPs will be recorded as a control to assess effects that are not specific for nociception [32]. The AEPs will be elicited by short-lasting auditory stimuli presented binaurally at a comfortable listening level (see separate operational manual). Such as for PEPs and LEPs, the auditory stimuli will be repeated 15 times in each block, with a randomly varying inter-stimulus interval. Such stimuli, delivered using relatively long and variable inter-stimulus intervals, are expected to generate a late-latency negative-positive complex (N1-P2), maximal at the scalp vertex, related to brain functions that are, at least in part, similar to those underlying the LEP and PEP N2-P2 response [37]. For each recording block, the amplitude of the N1-P2 will be measured as detailed in the operational manual.

#### Non-EEG/ERP-derived pharmacodynamic data

##### Pain intensity and unpleasantness ratings

Participants will be asked to rate the intensity of the sensation elicited by each laser and pinprick stimuli delivered to obtain the above-described EEG biomarkers, using a 101-point pain rating scale where 0 is defined as “no pain at all” and 100 is defined as “most intense pain imaginable” (detailed in a separate operational manual). These ratings, obtained after each stimulus, will allow changes in EEG biomarker measures to be related to changes in perception. Medication, induction of hyperalgesia, and/or effects of the medication on hyperalgesia may affect the intensity of the percept elicited by the different stimuli.

Furthermore, at the end of each laser and pinprick stimulation blocks, the participants will be asked to rate the average unpleasantness of the preceding series of stimuli that were delivered in that block using a 101-point unpleasantness rating scale where 0 is defined as “not unpleasant at all” and 100 is defined as “extremely unpleasant.”

Average pain ratings and unpleasantness ratings obtained at each PD assessment time-point and the results of the hyperalgesia testing will be calculated and entered in the source paper documentation before entering in the electronic CRF. Furthermore, all individual pain ratings obtained after each stimulus, all unpleasantness ratings obtained after each stimulation block, and the results of the hyperalgesia testing will be sent and stored centrally at UCLouvain, along with the pharmacodynamic data.

##### Patient-reported outcome measures (PROMs)

During the screening visit, participants will be asked to complete a set of PROMs assessing their psychological traits. During each study period, participants will be asked to complete an additional set of PROMs assessing their expectations, tiredness, and anxiety.

The following PROMs will be completed during the screening visit, in the following order:

1. *Self-assessment of general health using the PROMIS Global-10 questionnaire.* The PROMIS Global-10 is a publicly available global health assessment tool that allows measurements of symptoms, functioning, and healthcare-related quality of life for a wide variety of chronic diseases and conditions. It consists of 10 items that assess general domains of health and functioning including overall physical health, mental health, social health, pain, fatigue, and overall perceived quality of life.
2. *Self-assessment of self-efficacy using 3 items of the General Self-Efficacy Scale (GSE)*, followed by a short break. The GSE [38] is a psychometric scale that is designed to assess optimistic self-beliefs to cope with a variety of difficult demands in life.
3. *Self-assessment of trait anxiety using the General Anxiety Disorder-7 questionnaire (GAD-7)*. The GAD-7 is a brief scale on anxiety. This easy-to-use self-administered patient questionnaire is used as a screening tool and severity measure for generalized anxiety disorder.
4. *Self-assessment of depression using the Patient Health Questionnaire-9 (PHQ-9)*, followed by a short break. The Patient Health Questionnaire (PHQ) is a diagnostic tool for mental health disorders used by health care professionals that is quick and easy to complete. The PHQ-9 is a tool specific to depression and simply scores each of the 9 criteria based on the mood module from the original PRIME-MD, a diagnostic tool containing modules on 12 different mental health disorders.
5. *Self-assessment of pain catastrophizing using the Pain Catastrophizing Scale (PCS)* [39], followed by a short break. The PCS is a 13-item scale, with each item rated on a 5-point scale: 0 (not at all) to 4 (all the time). The PCS is broken into three subscales being magnification, rumination, and helplessness. The scale was developed as a self-report measurement tool that provides a valid index of catastrophizing in clinical and non-clinical populations. The results of the initial development and validation studies indicate that the PCS is a reliable and valid measurement tool for catastrophizing.
6. *Self-assessment of pain sensitivity using the Pain Sensitivity Questionnaire (PSQ))* [40]. The questionnaire contains a series of 17 questions in which the subject is asked to imagine himself in certain situations. He should then decide if these situations would be painful and how painful they would be. The PSQ uses a numeric rating scale from 0 = not at all painful to 10 = most severe pain imaginable.

The following PROMs assessing subject expectations will be completed at the beginning of each study period:

1. *Anxiety* will be assessed by asking participants: “On a scale of 0–100, please rate, how anxious you are about the upcoming experiment, with 0 being ‘not anxious at all’ and 100 being ‘extremely anxious’.”
2. *Pain expectation* will be assessed by asking participants: “On a scale of 0–100, please rate how much pain do you anticipate experiencing during the upcoming experiment, with 0 being ‘no pain at all’ and 100 being ‘pain as bad as you can imagine’.”
3. *Expectation of IMP-induced pain relief* will be assessed by asking participants: “On a scale of 0–100, please rate how much pain relief you expect from the medication, with 0 being ‘expecting no relief’ and 100 being ‘expecting complete relief’.”

The following PROMs assessing tiredness and anxiety will be assessed 5 h after IMP administration:

1. *Self-assessment of tiredness*. The subject will be asked: “On a scale of 0–100, please rate, how alert or sleepy you feel right now, with 0 being ‘very alert’ and 100 being ‘very sleepy’.”
2. *Self-assessment of state anxiety using the State and Trait Anxiety Inventory (STAI-Y).* The State-Trait Anxiety Inventory (STAI, by Mind Garden, Inc.©) is a psychological inventory based on a 4-point Likert scale and consists of 2 × 20 questions on a self-report basis [41]. The STAI-Y measures two types of anxiety state anxiety, or anxiety about an event, and trait anxiety (20 items), and anxiety level as a personal trait characteristic (20 items). Only state anxiety will be measured using this questionnaire. Higher scores are positively correlated with higher levels of anxiety. Its most current revision is Form Y. It is used in diagnoses, in clinical and other medical settings, as well as in research and is differentiating between anxiety and depression.

The PROMs will be collected via questionnaires in the local language. Scores calculated from these questionnaires will be entered in the source paper documentation before entering into the electronic CRF as described above. The subjects will be instructed on how to fill out the questionnaires. No queries will be issued to the investigator for these data, except for clarification of subject identifiers and any operational issues. All PROMs reported on paper will stay as source data at the trial site. The investigator will be responsible for the accurate transfer of these data into the electronic CRFs.

#### Hyperalgesia testing

The intensity of the sensation elicited by calibrated mechanical pinprick stimuli will be assessed at the left (HFS-sensitized) and right (non-sensitized) forearms. At the sensitized forearm, the extent of the area of secondary hyperalgesia will be measured by locating the position at which participants report a change in the intensity of the sensation elicited by mechanical pinprick stimuli delivered along 8 radial directions relative to where HFS was applied on the skin (proximal, proximal-lateral, lateral, lateral-distal, distal, distal-medial, medial, and medial-proximal directions). The same approach will be used to assess the intensity and area of dynamic allodynia (if present), using a standardized brush to deliver dynamic tactile stimuli. These assessments will allow the perceptual changes observed in IMI2-PainCare-BioPain-RCT3 to be compared to those observed in the other three RCTs of the BioPain subtopic of the PainCare project (IMI2-PainCare-BioPain-RCT1, 2, and 4).

### Plans to promote participant retention and complete follow-up {18b}

To promote participant retention, at the screening visit and at the four study periods, some exclusion criteria have been defined as temporary exclusion criteria, such as transient illness, changes in physical exercise, and medications taken in the past 2 weeks.

If at screening one or more of these temporary exclusion criteria are met, the subject can be included in the study. In this case, before the start of the first study period, the met temporary exclusion criteria of the screening visit will be checked again, and their absence will be verified before the first study period takes place.

Similarly, if screening of exclusion criteria for eligibility during a study period shows that one or more temporary exclusion criteria are met, the start of the study period may be postponed and re-scheduled. In this case, the previously met temporary exclusion criteria of the study period will be checked again and their absence will be verified before the subject is eligible for the period.

### Data management {19}

Data management will be performed by the Interdisciplinary Center for Scientific Computing at University Heidelberg. Documentation of the responsibilities and delegation thereof will be maintained in the trial master file.

All aspects of the data management process (including data validation and query management, medical coding, ECG data, biomarker data) and data lock procedures are described below and detailed in the data management plan.

#### Source data

All source data arising from the trial will be kept by the investigator, who will provide direct access for trial-related monitoring, audits, ethics committee review, and regulatory inspection.

#### Case report forms

Case report forms for each subject will be provided to the investigator in electronic format and will serve to create the local source documentation in paper format. The investigator and personnel delegated the task will use these paper-form documents to record the source data information required by the protocol. The source data documentation will be entered into a validated electronic CRF system via a secured access to the Heidelberg University computing center. The collected data will reside on secure servers of the Heidelberg University. Entry, corrections, and alterations of data within the system can only be performed by the investigator or other authorized personnel under their supervision and will be captured by the system’s audit trail. Entries will be checked against appropriate source documents by authorized CRAs as deemed appropriate in the monitoring guidelines. Dedicated users (e.g., the investigator, designated persons at the trial site, authorized sponsor representatives, and from other parties involved, e.g., data management) will be trained and receive access rights according to their role in the trial. All users will have access to the system and be able to review their data on an ongoing basis. After completion of the subject’s CRF, the CRF will be signed electronically by the investigator. With the investigators signature, it is confirmed that the data in the CRF are checked, complete, accurate, and in alignment with the source data. Changes to the CRF after initial signing by the investigator will need re-signing. With database lock, the edit rights to the CRFs will be removed, but the investigator will retain access to view the CRFs. The nature and location of all source data/clinical documentation will be identified and documented by the investigator to ensure that all sources of original data required to complete the CRF are known to the sponsor and/or trial site personnel and are accessible for verification during trial-related monitoring, audits, relevant IEC review, and inspection(s). During trial conduct, the Interdisciplinary Center for Scientific Computing at University Heidelberg is responsible for data security related to the data captured in the CRF.

#### Pharmacodynamic data

Copies of the raw pharmacodynamic source data will be electronically transferred to UCLouvain. The data will be stored on secured servers, which have backup onto a second server located in a physically distinct room, also using a secured storage protocol. Signal processing of the raw data to extract the parameters of interest will also be done centrally at UCLouvain using the Letswave 6 matlab toolbox for the analysis of EEG data (http://letswave.org; UCLouvain; hosted on the Github repository). All primary, secondary, and exploratory EEG biomarker endpoints derived from these analyses, as well as the non-EEG biomarker endpoints will be transferred to the relational database hosted at the Heidelberg University computing center. The obtained endpoint parameters will be entered by UCLouvain into a relational database located at the Heidelberg University computing center.

#### Pharmacokinetic data

Pharmacokinetic concentrations and pharmacokinetic parameters measured by ESTEVE will be uploaded by ESTEVE to the relational database at the Heidelberg University computing center.

#### Investigator’s site file and the trial master file

At each site, the Principal Investigator will be responsible for the filing of all essential documents in an investigator’s site file. The sponsor will be responsible for the timely filing of all essential documents in the trial master file. As applicable, these files must be available at monitoring visits and during audits or regulatory inspections. After trial completion, the Principal Investigator will ensure that all source data/documentation related to the trial is recorded, handled, and stored in a way that allows its accurate reporting, interpretation, and verification. The Principal Investigator will take measures to prevent accidental or premature destruction of these documents.

The Principal Investigator will keep the investigator’s site file, the source data/documentation arising from the trial according to the prescribed record retention period in the country and/or according to the hospital policy.

### Confidentiality {27}

Subject trial data will be stored in a manner maintaining confidentiality in accordance with applicable regulatory requirements.

The source data transferred from each site to UCLouvain for extraction of biomarker parameters, as well as the CRF data, will not contain any data that can identify the individual persons (such as name and social security number) except for a code, i.e., the data will be pseudoanonymized.

The investigator will ensure that any documents or data given to the sponsor or authorized sponsor representatives do not contain information that would affect the confidentiality of the subject’s identity.

All subject-related data will be obtained as described in this protocol document. There is no need to examine, analyze, verify, nor reproduce any healthcare or other records of the trial subjects.

### Plans for collection, laboratory evaluation, and storage of biological specimens for genetic or molecular analysis in this trial/future use {33}

#### Bioanalytical assays

The analysis of drug levels at all the different sampling times will be performed on pharmacokinetic samples collected from trial subjects who will be randomized to receive an active treatment (tapentadol, pregabalin, or lacosamide). Placebo samples will be analyzed at a single time-point around Tmax of drugs.

Drug concentrations of tapentadol, pregabalin, and lacosamide will be analyzed by ESTEVE Bioanalysis and ADME Development Department using a validated method under GLP (Good Laboratory Practice). The three drugs will be identified and quantified using HPLC method with tandem mass spectrometric detection (LC-MS/MS). Developed assays will be fully validated in terms of selectivity, linearity, accuracy, precision, recovery, matrix effects, and stability using official guideline on bioanalytical methods. Full details of the different analytical methods used will be described in the respective bioanalytical method validation report, and drug levels quantified in human plasma samples will be also reported in the respective bioanalytical report.

#### Bioanalytical laboratory unblinding

The bioanalytical laboratory will be unblinded in order to analyze multiple time-points from the specific active treatments and single time-points from corresponding placebos. The randomization list will be sent to the Bioanalysis and ADME Development Department at ESTEVE PHARMACEUTICALS, S.A. (ESTEVE) in order to analyze plasma drug levels of the active treatments (tapentadol, pregabalin, and lacosamide; multiple time-points) and placebo (single time-point). These analyses will be conducted when the clinical study is finished but the dataset is not yet locked. Therefore, the bioanalytical laboratory will recode the subject’s numbers to generate blinded results so that the investigator and the sponsor can review the results and remain blinded.

#### Calculation of pharmacokinetic parameters

The individual plasma concentration/time profiles for each analyte (tapentadol, pregabalin, and lacosamide) will be presented using the actual sampling times whereas the mean plasma concentration/time profiles will be presented using the theoretical sampling times. The plasma concentration data will be presented graphically for each analyte in a number of ways using both a linear and log-linear scale.

For each compound, relevant PK parameters will be calculated by standard non-compartmental methods for those subjects with sufficient plasma concentration data using Phoenix 64® WinNonLin® (Version 6.3 or later) with a log-linear terminal phase assumption. All reported sampling time deviations will be taken into consideration for evaluation of PK parameters.

For all three drugs, whenever applicable, the following standard non-compartmental pharmacokinetic parameters and drug-exposure-related metrics will be estimated in each subject, including:

- *C*_max_: maximum plasma concentration.
- *t*_max_: time to reach maximum plasma concentration.
- λz: the terminal phase constant will be calculated by linear regression of the last phase of the curve (log concentration vs time).
- *t*_1/2_: terminal half-life will be determined with the expression *t*_1/2_ = 0.693/λz.
- AUC0-*t*: Area under the plasma concentration-time curve from time zero to last quantifiable concentration calculated by the linear and/or log trapezoidal rule.
- AUC0-*∞*: The area under the curve of plasma levels vs time from zero to infinite will be obtained with the expression AUC0-∞ = AUC0-*t* + Clast/λz, where Clast is the predicted plasma concentration at the last time measured.

A descriptive analysis will be provided for each derived PK parameter. Below limit of quantitation (BLQ) concentrations will be treated as zero for all statistical analyses.

Full details of the pharmacokinetic analysis and the corresponding statistical analysis of PK parameters will be described in the final report.

## Statistical methods

### Statistical methods for primary and secondary outcomes {20a}

This and the following sections briefly specify the statistical analysis principles for the study. Final and detailed specifications on the quantitative analyses described here will be provided correspondingly in the statistical analysis plan to be finalized prior to unblinding of the study.

The general level of significance used is *α* = 5% (two-sided). However, this will only be considered as confirmatory within the multiplicity adjusted statistical analysis of the primary endpoints and the key secondary endpoints.

#### Analysis sets

The “all enrolled set” includes all subjects enrolled into the study who have provided at least some data. The “full analysis set” consists of the subjects in the all enrolled set that have been randomized. The “safety set” includes all subjects of the full analysis set that have received at least one dose of any study medication. Further analysis populations (e.g., per protocol set) may be defined in the statistical analysis plan.

Unless otherwise specified in the respective sections below the following populations will be used for the analyses. Demographic and baseline characteristics analyses will be based on the all enrolled set and, optionally, the full analysis set or safety set. Efficacy data (primary and secondary endpoints) will be analyzed based on the full analysis set using the intent-to-treat principle, i.e., a subject will be assigned to the treatment group he or she was randomized to. Safety analyses will be based on the safety set. Should there be a huge discrepancy between some populations, such as safety set being less than 90% of all enrolled set, some analyses may additionally be conducted for the different analysis set.

#### Definition of the primary endpoints

The first primary endpoint is the percentage of change in amplitude of the negative-positive vertex potential (N2-P2 complex) of LEPs at time-point T + 60 min post-drug administration vs. the pre-drug time-point, at the non-sensitized arm. The second primary endpoint is the percentage of change in amplitude of the N2-P2 complex of PEPs at time-point T + 60 min post-drug administration vs. the pre-drug time-point, at the sensitized arm.

$$ \mathrm{endpoint}=\frac{\mathrm{N}2-\mathrm{P}{2}_{\mathrm{amp}}\left(+60\ \min \right)-\mathrm{N}2-\mathrm{P}{2}_{\mathrm{amp}}\left(\mathrm{baseline}\right)}{\mathrm{N}2-\mathrm{P}{2}_{\mathrm{amp}}\left(\mathrm{baseline}\right)} $$

The primary analysis of the two primary endpoints will focus on the comparison of the tapentadol vs. placebo effects.

Within the error-controlled testing procedure, both primary endpoints will also be tested in key secondary analyses comparing lacosamide vs. placebo and pregabalin vs. placebo effects. Also within the error-controlled testing procedure, the key secondary analyses will also compare a third endpoint: the percentage of change in amplitude of ongoing theta band EEG oscillations at time-point T + 60 min post-drug administration vs. the pre-drug time-point.

$$ \mathrm{endpoint}=\frac{{\mathrm{theta}}_{\mathrm{amp}}\left(+60\min \right)-{\mathrm{theta}}_{\mathrm{amp}}\left(\mathrm{baseline}\right)}{{\mathrm{theta}}_{\mathrm{amp}}\left(\mathrm{baseline}\right)} $$

#### Definition of secondary endpoints

The following secondary endpoints will be investigated:

- The percentage of change in the intensity of the sensation elicited by laser stimulation of the non-sensitized forearm at time-point T + 60 min post-drug administration vs. the pre-drug time-point.
- The percentage of change in the intensity of the sensation elicited by mechanical pinprick stimulation of the sensitized forearm at time-point T + 60 min post-drug administration vs. the pre-drug time-point.
  $$ \mathrm{endpoint}=\frac{{\mathrm{pain}}_{\mathrm{intensity}}\left(+60\min \right)-{\mathrm{pain}}_{\mathrm{intensity}}\left(\mathrm{baseline}\right)}{{\mathrm{pain}}_{\mathrm{intensity}}\left(\mathrm{baseline}\right)} $$

#### Statistical procedure

**Fig. 5:**
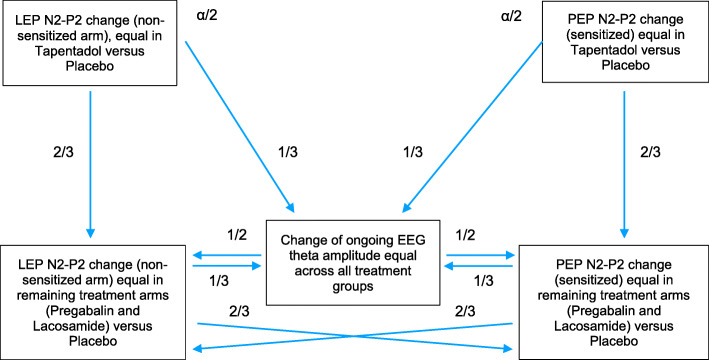
Statistical procedure. The sequentially rejective multiple testing approach used for the selected confirmatory analyses. The two primary endpoints (LEP N2-P2 and PEP-N2-P2) will be tested in parallel, splitting the overall *α* equally between the endpoint tests. If any of these two tests shows significant differences, key secondary tests will be conducted using the *α*-levels as passed on from initial/prior tests according to specified weights

The statistical procedure for the selected, confirmatory analyses will follow the sequentially rejective multiple testing approach described by Bretz et al. [42]. The two primary endpoints (LEP N2-P2 amplitude change at non-sensitized arm and PEP N2-P2 amplitude change at sensitized arm) will be tested for their differences between the treatment arms tapentadol versus placebo, first. This will be conducted in parallel, splitting the overall *α* equally between the two endpoint tests, i.e., each test has a type I error of *α*/2. If any of these two tests shows significant differences, key secondary analyses will be conducted using the *α*-levels as passed on from initial/prior tests according to specified weights. The exact procedure (with the local levels as well as the weights with which to pass *α*-levels on) is shown in Fig. 5, and related specifications are provided in the subsequent subsections.

#### Primary analysis of primary endpoints

The primary analyses consist of two parallel tests (based on full analysis set data) which differ exclusively in the endpoint LEP N2-P2 endpoint and PEP N2-P2 endpoint). Only the analysis for the LEP N2-P2 change endpoint is specified here. Analysis of the PEP N2-P2 change endpoint is identical and will be provided in the statistical analysis plan.

LEP N2-P2 change endpoint data being repeated measurements (at the T + 60 min time-point post-dose) across the four periods will be analyzed with a mixed effect model with treatment (4 levels), period (4 levels), center, and sequence as fixed effects. The variance-covariance structure for the repeated measure variable period should be chosen as CS (compound symmetry).

The LS means difference of treatment tapentadol versus placebo will be estimated and tested based on this model and the estimate as well as the corresponding unadjusted confidence interval and *p* value will be provided.

If either (or both) of the primary endpoints’ tests show significant differences between tapentadol and placebo in the sense that the *p* value is below the corresponding local alpha level, the differences are confirmed, and the testing procedure continues according to the graph above.

#### Key secondary analyses of primary endpoints

These statistical analyses are considered confirmatory only provided they have received local *α*-levels (type I error greater than 0) from prior tests rejecting their corresponding null hypotheses.

The method for testing the primary endpoints comparing the other treatment groups versus placebo mirrors the model specifications of the primary analysis above but using the Dunnett-adjusted estimation and testing results to compare pregabalin and lacosamide with placebo respectively.

The method for testing the key secondary endpoint theta band oscillations (theta_amp_) in ongoing EEG is similar to these specifications using the model of the primary analysis above but extending the Dunnett comparisons to all three drug treatment arms versus placebo.

#### Analysis of secondary endpoints

All secondary endpoints will be compared across the four treatment groups using the same approach as the analysis of the primary endpoints. These analyses, even if using the general significance level above, are considered exploratory.

### Interim analyses {21b}

No interim analysis is foreseen for this study. Should it later become necessary to conduct an interim analysis, it will be described in an amendment to this protocol prior to conduct of the analyses. The amendments will include considerations on maintaining the blind as well as specifications of appropriate statistical adjustments for unplanned interim analyses.

### Methods for additional analyses (e.g., subgroup analyses) {20b}

There are many other analyses corresponding to the additional time-points when assessments are done (180 and 360 min post-dose), but also by the collection of other biomarkers, i.e., other outcomes. A selection of these is briefly described below.

- Latency of the N2 and P2 components of LEPs and PEPs. In addition to affecting the amplitude of the N2-P2 complex of LEPs and PEPs, the different drugs could also affect the latency of laser- and/or pinprick-evoked brain responses, via effects on peripheral and/or central conduction times. We will thus examine whether the latencies of the N2 and P2 components of LEPs and PEPs are sensitive to drug effects.
- Early-latency N1 component of LEPs. In addition to the N2-P2 complex, laser stimuli also elicit an early-latency negative wave, referred to as the N1 component, and maximal at central-temporal electrodes contralateral to the stimulated upper limb. This component is thought to reflect earlier stages of nociceptive processing, primarily originating from the contralateral operculo-insular cortex and/or the primary somatosensory cortex. We will thus examine whether the amplitude and/or latency of this N1 component is sensitive to drug effects.
- Dimensionality reduction and feature selection methods. Spatio-temporal or spatio-spectral blind source separation techniques such as independent component analysis [43], microstate EEG analysis [44], or extensions of this analysis technique currently developed by UCLouvain can be used to reduce the dimensionality of multichannel ongoing EEG as well as pain-evoked brain responses to identify EEG features that may be sensitive to drug effect on nociception and/or its interaction with sensitization.
- Single-trial ERP amplitude/latency dispersion measures. Single-trial estimates of the amplitude of LEP N2-P2 amplitude and PEP N2-P2 amplitude can be obtained using methods developed in part by UCLouvain [45]. These estimates can be used to examine whether drug administration affects the trial-to-trial variability in ERP amplitude or latency.
- Cluster-based point-by-point LEP and PEP waveform analysis. Rather than restricting the analysis of LEP and PEP waveforms to the estimation of amplitude and latency of N2 and P2 peaks, a point-by-point waveform analysis can be used to compare the entire waveform of LEPs and PEPs across conditions. This approach, combined with cluster-based permutation testing to address the issue of multiple comparisons, has been used successfully to identify changes in PEPs induced by HFS [5, 46].
- Topographical analysis of LEPs and PEPs, and source waveform estimation for primary and secondary somatosensory cortex, insula, anterior cingulate cortex, and prefrontal cortex activation. Because the different drugs may differentially affect the different cortical sources of LEPs and PEPs, the topographical distribution of the N1, N2, and P2 components of PEPs and of the N2 and P2 components of PEPs will be compared across the different treatment groups. Furthermore, source waveform estimation of laser- and pinprick-evoked activity in the primary and secondary somatosensory cortex, the insula, the anterior cingulate cortex, and the prefrontal cortex can be obtained using distributed source modeling (e.g., sLORETA algorithm) [47]. This can be used to assess selective changes in the magnitude of the activity generated within these different structures.
- Ongoing EEG oscillations. In addition to drug effect on the magnitude of theta band EEG oscillations, we will also assess potential drug effects on delta-, alpha-, beta-, and gamma-band oscillations, as these could be differentially modulated by the different treatments. This will include analysis of cross-frequency coupling, and analysis of across-channel phase coherence. Furthermore, previous studies have shown that both laser stimuli and pinprick stimuli induce transient increases and decreases in the magnitude of these ongoing EEG oscillations, referred to as event-related synchronization (ERS) and desynchronization (ERD) [5, 36, 48]. Therefore, we will examine whether the different treatments affect laser- and pinprick-induced ERD and ERS.

#### Pharmacometric analyses

The data collected in this trial together with the data from the other three IMI2-PainCare-BioPain-RCTs and the preclinical studies of the BioPain project will be subject to pharmacometric analyses with the intention to validate biomarkers that can translate from preclinical to clinical readouts. As both drug concentrations and biomarker responses are measured at several time-points post-drug administration, the relationship between drug levels and select biomarkers will be explored and modeled. The pharmacometric analysis will be described in a separate pharmacometric analysis plan and will consist briefly in developing population pharmacokinetic/pharmacokinetic (PK/PD) models estimating the primary PK parameters (i.e., apparent volume of distribution, and total plasma clearance), the primary PD parameters (i.e., C50, the plasma or effect site concentration that elicits a response equal to half of the maximum attainable effect (EMAX)), and their associated inter-individual variability.

#### Analysis of demographic data and other baseline characteristics

Analyses of demographic data and other baseline characteristics will consist of descriptive summary statistics. While these tables may provide the summaries by groups (i.e., treatment sequences), there will be no statistical tests conducted on baseline characteristics differences. Data will also be listed. Details will be provided in the statistical analysis plan.

#### Analysis of test-retest reliability

A reliability analysis will investigate the biomarker and their measurement characteristics using the following concepts, all based on the full analysis set. Detailed specifications will be provided in the statistical analysis plan. Assessment of the repeatability of a (individual) biomarker response measurement method will use the statistics described by Bland and Altman [49] (such as differences against means plots). Also, within- and between-subject variabilities will be estimated using the mixed AN(C) OVA models including the corresponding variance components according to the repeated measurements design. These include data across time-points within a “visit” (e.g., during placebo treatment period) or across “visits” (e.g., baseline measurements across the treatments periods) as applicable. Should measurement methods of the same biomarker be compared, the agreement statistics of Bland and Altman will be used. Assessment of the reproducibility will follow the statistics (such as difference limits, minimum significant difference, limits of agreement) as described in standard guidance documents. As applicable, further statistics presented for test-retest characteristics may include correlations (such as concordance correlation coefficients, intraclass correlation coefficients), coefficients of variations, and sensitivity to change statistics, e.g., smallest real difference [50].

Any changes to the planned statistical analyses, if considered prior to unblinding, will be described and justified in an amendment to the protocol (if study is still ongoing) and to the statistical analysis plan. Deviations from planned analyses will also be explained in the final study report.

### Methods in analysis to handle protocol non-adherence and any statistical methods to handle missing data {20c}

The analysis method of the mixed effect model already takes missing data into account and missing data issues are also partly addressed by the sensitivity analyses. More thorough and complete descriptions of missing data handling will be provided in the statistical analysis plan.

### Plans to give access to the full protocol, participant level-data, and statistical code {31c}

This will be determined at the end of the project period of IMI-PainCare.

## Oversight and monitoring

### Composition of the coordinating center and trial steering committee {5d}

This trial is part of the Biopain subtopic of the IMI2 PainCare project.

The PainCare Innovative Medicines Initiative (www.imi-paincare.eu) is a partnership between the European Union and the European pharmaceutical industry. IMI facilitates open collaboration in research to advance the development of and accelerate patient access to personalized medicines for the health and well-being of all, especially in areas of unmet medical need. The IMI-PainCare Consortium is composed of 40 participants from 14 countries; 6 are EFPIA (European Federation of Pharmaceutical Industries and Associations) participants with experience in pain research and development, 23 are academic and clinical institutions, 5 are specialist SMEs, 3 are patient organizations, and 3 are professional pain/anesthesia societies.

#### Project coordinator

The overall coordination of the IMI-PainCare project is performed by the Project Coordinator who acts simultaneously as Co-Lead for the subproject BioPain including the IMI2-PainCare-BioPain RCTs (Prof. Rolf-Detlef Treede, Heidelberg University, Germany).

#### RCT Lead

The RCT Lead will act as the international coordinating investigator who is responsible for the coordination of the Principal Investigators at multiple trial sites in multiple countries. The RCT Lead will be the sponsor of the study (Prof. André Mouraux, Université Catholique de Louvain, Belgium).

#### Investigators

There will be one Principal Investigator at each trial site. If, at the trial site, the trial is conducted by a team of individuals, the investigator leading and responsible for the team is called the Principal Investigator, and the other individuals of the team are called investigators. Curriculum vitae and/or other relevant documents confirming the current qualification of the investigators will be provided to the sponsor. This includes any previous training in the principles of GCP, experience obtained from work with clinical trials, and experience with subject care. Documentation of all involved investigators will be maintained according to GCP and applicable regulatory requirements. In different countries, there may be country-specific terminology used for the investigator role.

#### Trial site personnel assigned trial-related duties

The Principal Investigator can define appropriately qualified personnel at a trial site to perform significant trial-related procedures and/or to make trial-related decisions under his/her supervision. In this case, the Principal Investigator will maintain a signed list of the persons to whom they delegate significant trial-related duties/responsibilities; the delegated trial-related duties/responsibilities will be specified in the list. When personnel or responsibility changes are made, the Principal Investigator will ensure that the relevant documentation is updated before any trial-related activities are performed. Documentation of all involved trial site personnel performing significant trial-related procedures and/or making trial-related decisions will be maintained according to GCP and applicable regulatory requirements.

#### External ethics advisory board

The IMI-PainCare project has been approved under the condition that the consortium carrying out the project implements an external ethics advisory board which (1) reviews the proper application of the relevant laws and guidelines containing ethical rules and the H2020 rules by the investigators; (2) provides advice to and monitors the activities of the investigators with regard to ethical issues; and (3) provides advice on the compliance with European ethical laws and regulations and with different guidelines, laws, and regulations of countries where studies are being performed. For practical implementation, ConsulTech, non-clinical partner of the study, will collect all relevant ethical documents and will ensure that all partners submit them on time. ConsulTech will then draw up a questionnaire for the ethics advisory board, which will allow it to check whether all important ethical requirements and documents have been submitted and that all legal guidelines have been adhered to.

### Composition of the data monitoring committee, its role and reporting structure {21a}

A data monitoring committee is not foreseen. Data management will be performed by the Interdisciplinary Center for Scientific Computing at University Heidelberg. Documentation of the responsibilities and delegation thereof will be maintained in the trial master file. All aspects of the data management process (including data validation and query management, medical coding, ECG data, biomarker data) and data lock procedures are described in the data management plan.

### Adverse event reporting and harms {22}

Adverse events will be documented from the time of enrollment (i.e., the time the informed consent form is signed) up to the time of the last protocol scheduled contact, i.e., date of last visit/contact (can be a phone call, e.g., in case of withdrawal). Subjects in the trial have the opportunity to report adverse events spontaneously. There will also be given a general incitement for events, such as “Have you noticed anything unusual about your health (since last visit)?”. In addition, the investigator will go through all self-assessed procedures (e.g., patient-reported outcomes and questionnaires) used in the trial. Present conditions on entry into the study shall be registered in the medical history, if applicable. Date of onset and termination for each adverse event, and impact of the test substance will be registered. The severity of the adverse event and the relationship between the treatment drug and the adverse event will be assessed (see below).

#### Definitions

- Adverse event (AE): any untoward medical occurrence in the subject or clinical trial subject administered a medicinal product and which does not necessarily have a causal relationship with this treatment.
- Adverse reaction (AR): any untoward and unintended response to an investigational medicinal product related to any dose administered.
- Unexpected adverse reaction (UAR): an adverse reaction, the nature or severity of which is not consistent with the applicable SmPC is used as the reference safety information to assess whether a serious adverse reaction (SAR) is considered to be expected or unexpected.
- Serious adverse event (SAE) or serious adverse reaction (SAR): an untoward medical occurrence or affect that at any dose results in death, is life-threatening, requires hospitalization or prolongation of existing hospitalization, results in persistent or significant disability, or is a congenital anomaly or birth defect.
- Suspected unexpected serious adverse reaction (SUSAR): an untoward and unintended response to a study drug, which is not listed is the applicable product information, and meets one of the following serious criteria: results in death, is life-threatening, requires hospitalization or prolongation of an existing hospitalization, results in persistent or significant disability or incapacity, or is a congenital anomaly or birth defect.

#### Guideline for assessing the relationship between adverse events and treatment

- Unrelated—no temporal relationship or other etiologies are very likely the cause.
- Unlikely related—unlikely temporal relationship or other etiologies are more likely the cause.
- Possibly related—less clear temporal relationship, other etiologies are also possible.
- Probably related—clear temporal relationship with improvement after discontinuation of medicament and not reasonably explained by the subject’s clinical condition known.
- Related—clear temporal relationship with laboratory confirmation or a positive new additional treatment trial.

#### Severity grading

- Mild—usual transient and generally not interfering with normal activities.
- Moderate—discomforting enough to interfere with normal activities.
- Severe—prevents normal activities.

##### Reporting of adverse events

Investigators will report all SAEs to the sponsor as soon as possible and within 24 h. This in order for the sponsor to be able to report to the competent authority (CA) and the IEC if it is considered a SUSAR. The information to the sponsor will include subject number, name of investigator, detailed description of the SAE, investigator’s assessment of the relationship between the AE and the study drug, a statement that the event is serious and that it is unexpected, and the consequence for the study and the serious adverse event form.

The sponsor will ensure that information about all unexpected and serious adverse reactions, which are fatal or life-threatening, are registered and reported to CA and IEC as soon as possible and not later than 7 days after the sponsor has been made aware of such a suspected adverse reaction. Not later than 8 days after the reporting, the sponsor will notify the CA and the IEC with all relevant information on sponsor’s and investigator’s follow-up on the report. All other unexpected and serious suspected adverse reactions will be reported to CA and IEC not later than 15 days after the sponsor has been made aware of these. Reporting will be done electronically on the SUSAR’s form for the reporting for non-commercial researchers to the CA, and the same form can be printed and sent out in PDF format to the ethical committee. The guidelines from the CA for reporting of adverse reactions in clinical trials will be followed.

During the entire trial duration, the sponsor will provide all information needed to the local ethics committees and competent agencies (Belgian Federal Agency for Medicines and Health products FAMHP).

Once a year during the entire trial period, the sponsor will prepare a list of all suspected SAEs, which have occurred during the trial period, and a report on the trial subjects’ safety. List and report will be submitted to the CA and the IEC.

All adverse events shall be reported at the end of the trial in the final registration of trial results in EudraCT and in the report to the ethical committee if requested.

##### Follow-up after reporting of an adverse reaction

Following the adverse effect reported by the subject, this will be followed up at the next visit. If the subject drops out of the trial, the adverse event will be followed up with a telephone call after 1 month.

##### Reference document

The national SmPC is used as a reference document for assessing SUSARs.

##### Subject’s dropout because of events

Any subject can as a result of an event, regardless of the time, be excluded from the trial according to the investigator’s discretion.

### Frequency and plans for auditing trial conduct {23}

The non-clinical partner Consultech will coordinate trial monitoring activities. Trial site monitoring as defined in GCP will be performed by authorized personnel of a subcontracted CRO at pre-defined intervals and according to a monitoring plan depending on the progress of the trial. Corrections, amendments, or clarifying statements resulting from monitoring visits will be made by the investigator where necessary. Appropriate checking against source documents will be done.

#### Inspections

The Principal Investigator, any investigator(s), the sponsor, or personnel at other establishments, will cooperate with any inspection of the documents, facilities, records, and other resources deemed appropriate by the inspecting authorities to be related to the trial and that may be located at the trial site, at the sponsor, or at other establishments.

### Plans for communicating important protocol amendments to relevant parties (e.g., trial participants, ethical committees) {25}

Any modifications to the protocol which may impact on the conduct of the study, potential benefit of the patient, or may affect patient safety, including changes of study objectives, study design, patient population, sample sizes, study procedures, or significant administrative aspects will require a formal amendment to the protocol. Such amendment will be approved by the IEC and prior to implementation and notified to the health authorities in accordance with local regulations.

If changes to the trial are implemented after the initial insurance was arranged, e.g., due to protocol amendments, the Principal Investigator will notify the insurance company of these changes in accordance with the insurance conditions. If changes to insurance arise, the Principal Investigator will inform the subjects at his trial site about relevant changes.

Subjects will be informed as soon as possible if new information becomes available that may be relevant to their willingness to continue participation in the trial. The communication of this information will be documented.

### Dissemination plans {31a}

A final report integrating trial results will be prepared. The Principal Investigator will provide the competent authority/ies and relevant IEC(s) with a summary of the trial results in accordance with applicable regulatory requirements.

The results of this trial will be publicly disclosed (EudraCT). The results (or parts thereof) of this trial will be published according to the Grant Agreement and Consortium Agreement of IMI-PainCare (grant No 777500).

## Discussion

IMI2-PainCare-BioPain-RCT3 is one of four RCTs aiming at validating biomarkers of drug effects on nociceptive processing using the same trial design and IMPs. In addition to the primary efficacy analysis, we will conduct exploratory data analyses aimed to elucidate the inter-relationships between all endpoints investigated in this trial and the other trials. If there is evidence for a relationship among the different biomarkers, in part induced by a common underlying mechanism, we will seek to establish a latent variable measurement model aimed to quantify the underlying mechanism. This latent variable might be interpreted as a summary score aggregating the information of multiple markers.

In more detail, the hierarchical modeling will aim to:

- Elucidate interdependence pattern among potentially correlated markers.
- Establish measurement models for quantifying latent constructs related to the pain compartments of interest that might better reflect the causal mechanisms of treatment effect.
- If there is more than one latent variable identified, fit a structural model that specifies the (inter) dependencies between latent compartment structures as well as the efficacy pathways of the different treatment modalities.

Finally, a synthesis of the findings of the hierarchical modeling of this trial with the findings from the other three IMI2-PainCare-BioPain-RCTs will help to better understand how the efficacy pathways of different treatments with respect to different compartments are inter-related.

At the end of the project period, a proposal will be drafted describing how a subset of the validated functional pain biomarkers may be included in a Phase II RCT design in patients.

## Trial status

This manuscript is based on protocol version 3.0 dated 15/05/2019. Recruitment started 01/09/2020 and is expected to end before 31/12/2021.

## Notes

1. EudraCT 2019–000942-36; https://clinicaltrialsregister.eu/ctr-search/trial/2019-000942-36/DK
2. EudraCT 2019–000755-14
3. EudraCT 2019–001204-37; https://clinicaltrialsregister.eu/ctr-search/trial/2019-001204-37/BE
4. EudraCT 2019–000908-15; https://clinicaltrialsregister.eu/ctr-search/trial/2019-000908-15/DK

## Abbreviations

AE: Adverse event
AV: Atrioventricular
AEP: Auditory-evoked potential
AR: Adverse reaction
BID: Bis in die: twice daily
CA: Competent authority
*C*_max_: Peak concentration
CNS: Central nervous system
CRF: Case report form
IEC: Independent Ethics Committee
ECG: Electrocardiography or electrocardiogram
EEG: Electroencephalography or electroencephalogram
EFPIA: European Federation of Pharmaceutical Industry Associations
EMA: European Medicines Agency
EU: European Union
GCP: Good Clinical Practice
ICH: International Conference on Harmonization
IMI: Innovative Medicines Initiative (of the EU)
IMP: Investigational medicinal product (see “Definition of terms”)
K2-EDTA: Dipotassium ethylenediaminetetraacetic acid
LEP: Laser-evoked potential
N: Number (of subjects)
N2-P2: negative (N2) - positive (P2) complex of LEPs and PEPs
PD: Pharmacodynamics
PEP: Pinprick-evoked potential
PI: Principal Investigator
PK: Pharmacokinetics
PK-PD: Pharmacokinetics-pharmacodynamics
PR: Interval of the ECG
PROM: Patient-reported outcome measure
RCT: Randomized controlled trial
SAE: Serious adverse event
SD: Standard deviation
SmPC: Summary of Product Characteristics
SUSAR: Suspected unexpected serious adverse reaction
UAR: Unexpected adverse reaction
*t*_1/2_: Half-life
*t*_max_: Time to reach peak concentration
UCB: Union Chimique de Belgique, a pharmaceutical company
